# Multilineage contribution of CD34^+^ cells in cardiac remodeling after ischemia/reperfusion injury

**DOI:** 10.1007/s00395-023-00981-8

**Published:** 2023-05-05

**Authors:** Jun Xie, Liujun Jiang, Junzhuo Wang, Yong Yin, Ruilin Wang, Luping Du, Ting Chen, Zhichao Ni, Shuaihua Qiao, Hui Gong, Biao Xu, Qingbo Xu

**Affiliations:** 1 Department of Cardiology, Drum Tower Hospital, Affiliated Hospital of Medical School, Nanjing Universityrsity, State Key Laboratory of Pharmaceutical Biotechnology, No. 321 Zhongshan Road, Nanjing, 210008 Jiangsu People’s Republic of China; 2 Department of Cardiology, The First Affiliated Hospital, Zhejiang University School of Medicine, 79 Qingchun Road, Hangzhou, 310003 Zhejiang Province People’s Republic of China; 3 Alibaba-Zhejiang University Joint Research Center of Future Digital Healthcare, Hangzhou, People’s Republic of China

**Keywords:** Myocardial ischemia/reperfusion injury, Single-cell RNA sequencing, Mesenchymal cell, Endothelial cell, Macrophage

## Abstract

The ambiguous results of multiple CD34^+^ cell-based therapeutic trials for patients with heart disease have halted the large-scale application of stem/progenitor cell treatment. This study aimed to delineate the biological functions of heterogenous CD34^+^ cell populations and investigate the net effect of CD34^+^ cell intervention on cardiac remodeling. We confirmed, by combining single-cell RNA sequencing on human and mouse ischemic hearts and an inducible *Cd34* lineage-tracing mouse model, that *Cd34*^+^ cells mainly contributed to the commitment of mesenchymal cells, endothelial cells (ECs), and monocytes/macrophages during heart remodeling with distinct pathological functions. The *Cd34*^+^-lineage-activated mesenchymal cells were responsible for cardiac fibrosis, while CD34^+^Sca-1^high^ was an active precursor and intercellular player that facilitated *Cd34*^+^-lineage angiogenic EC-induced postinjury vessel development. We found through bone marrow transplantation that bone marrow-derived CD34^+^ cells only accounted for inflammatory response. We confirmed using a *Cd34*-CreER^T2^; R26-DTA mouse model that the depletion of *Cd34*^+^ cells could alleviate the severity of ventricular fibrosis after ischemia/reperfusion (I/R) injury with improved cardiac function. This study provided a transcriptional and cellular landscape of CD34^+^ cells in normal and ischemic hearts and illustrated that the heterogeneous population of *Cd34*^+^ cell-derived cells served as crucial contributors to cardiac remodeling and function after the I/R injury, with their capacity to generate diverse cellular lineages.

**Supplementary Information:**

The online version contains supplementary material available at 10.1007/s00395-023-00981-8.

## Introduction

Although primary revascularization therapy is the standard treatment for ST-segment elevation myocardial infarction (MI) to restore myocardial function, additional cardiac damage induced by reperfusion [ischemia/reperfusion (I/R) injury] still occurs, leading to further cardiac deterioration [11, 12]. However, the impact of different cell types in this process has not been fully elucidated. CD34 is a widely expressed cell-surface marker in several stem/progenitor cells, including hematopoietic stem cells and endothelial progenitor cells (EPCs) [31], and many studies have reported on CD34^+^ stem cell therapy. Although this strategy seems promising in some clinical trials with benefits (reduced infarct size and collagen deposition, and increased capillary density) [28], several studies failed to achieve an improvement in cardiac function in the long-term observation [23]. Subsequent work in clinical studies suggested that only 1.3–2.6% of infused stem cells were retained in the heart [29]. The ambiguous results have made the identification of the exact identity and function of these cells challenging. Whether CD34^+^ cells actually participate in cardiac repair has remained controversial for decades. On the contrary, recent studies indicated the presence of tissue-resident CD34^+^ cells with proliferating and differentiating capacity [13], but their exact role in myocardial I/R injury is still unknown.

## Materials and methods

A detailed description of the methods can be found in the Supplemental Materials.

### Human heart tissue collection

The human infarcted heart samples were collected from patients diagnosed with MI prior to heart transplantation. The control heart was collected from a generous healthy donor for another organ transplantation who gave consent for heart sample collection for research purpose. All samples were taken from left ventricles during the surgery. Written informed consent was obtained from the patients. All samples were collected from the Nanjing Drum Tower Hospital (China) or the First Affiliated Hospital of Zhejiang University (China) between September 2019 and December 2020. The baseline characteristics of patients are summarized in Table S6. All experiments were approved by the research ethics committees of the two aforementioned hospitals. All procedures had local ethical approval, and all experiments were conducted according to the 2013 Declaration of Helsinki [36].

### Ethics statement

All animal procedures conformed to the Guide for Care and Use of Laboratory Animals published by the US National Institutes of Health (NIH; 8th edition, 2011) and were approved by the Institutional Ethics Committee and performed according to the guidelines set by the Nanjing Drum Tower Hospital, China.

### Mouse generation and breeding

The mouse lines used in this study were as follows: *Cd34*-CreER^T2^ mice was a self-generated transgenic mouse line generously provided by Prof. Qingbo Xu’s Laboratory (Zhejiang University and Queen Mary University of London). The C57BL6/J, R26-tdTomato (JAX: 007909), and R26-eGFP-DTA (JAX: 006331) mouse lines were purchased from the Shanghai Biomodel Organism Co., Ltd. The *Cd34*-CreER^T2^;R26-tdTomato mice were obtained by crossing *Cd34*-CreER^T2^ with Rosa26-tdTomato mice. The *Cd34*-CreER^T2^;R26-DTA/tdTomato mice were obtained by crossing *Cd34*-CreER^T2^ mice with Rosa26-tdTomato mice and R26-eGFP-DTA mice. The tissue was lysed with proteinase K, precipitated using isopropanol, washed with 70% ethanol, and prepared for genotyping.

### Cardiac surgeries

MI or myocardial I/R model was established by treating wild-type mice or *Cd34*-CreER^T2^;R26-tdTomato mice, aged 8–12 weeks, with tamoxifen. The procedures were performed according to established previous studies [18].

### Statistical analyses

All data were determined from multiple individual biological samples and presented as mean values ± standard error of the mean. The “*n*” in the study represented the number of biological replicates, which is indicated in the figure legends. The GraphPad Prism 9 was used to perform the statistical analysis. The data were first analyzed for normality test, followed by the unpaired-sample Student’s *t* test or one-way analysis of variance test, which are indicated in the figure legends. A *P* value < 0.05 indicated a statistically significant difference. All mice were randomly assigned to different experimental groups.

## Results

### Distinct pathological characteristics of CD34^+^ cells in cardiac I/R injury

**Fig. 1:**
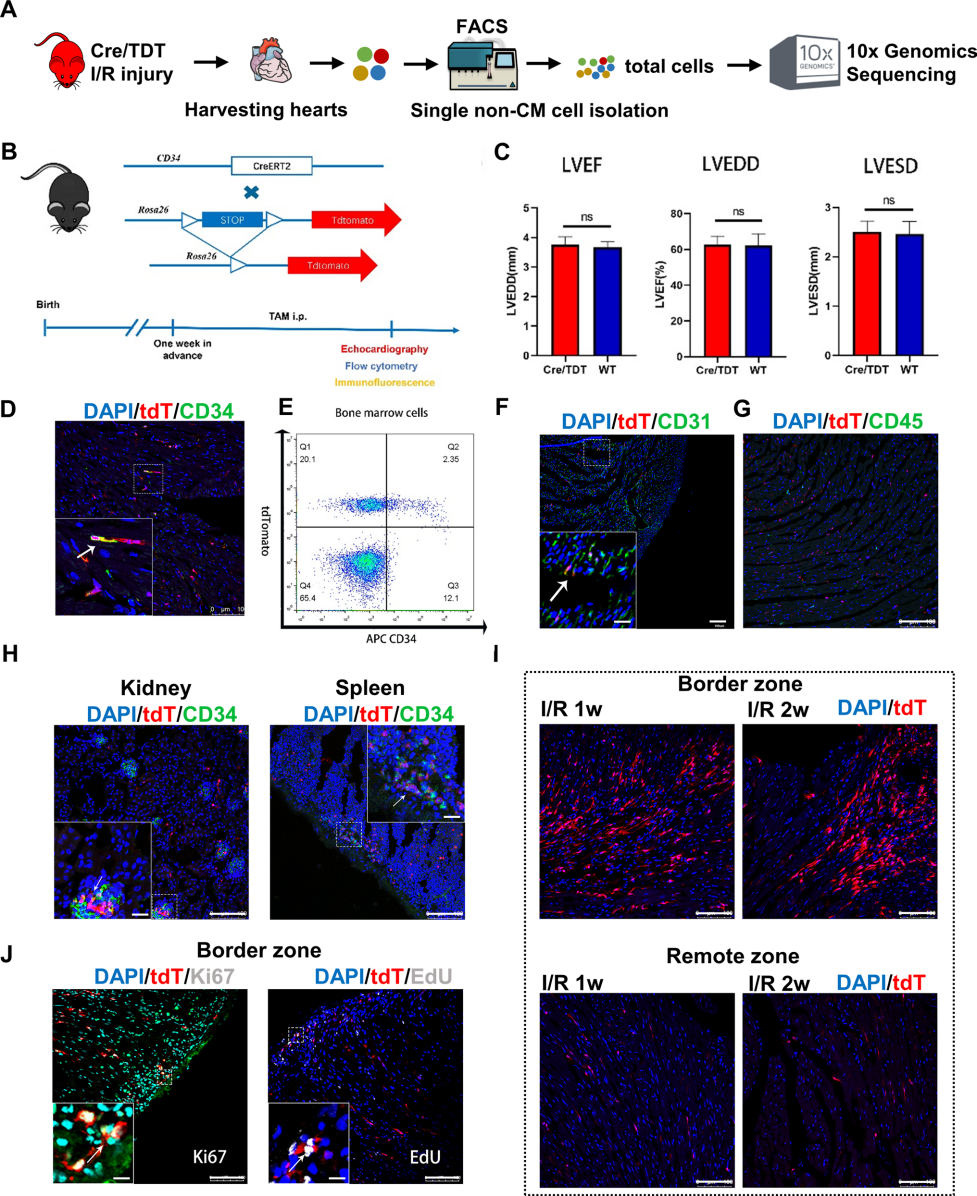
Active participation of tdTomato-labeled *Cd34*-lineage cells in I/R injury. **A** Schematic depicting heart tissue harvested from C57BL6 mice for scRNA-seq. The left anterior descending coronary artery (LAD) was temporarily ligated and reperfusion was conducted 45 min later. Sham surgery was performed by only opening the thoracic cavity without ligation. Heart tissue was harvested 1 and 2 weeks after surgery, along with hearts from the sham group. Single cells were then isolated from the left ventricle, sorted by fluorescence-activated cell sorting (FACS) and subjected to scRNA-seq (*n* = 3 mice per group). **B** The experimental scheme whereby *Cd34*-CreER^T2^; R26-tdTomato (Cre/TDT) mice were given tamoxifen 1 week before I/R or sham surgery. Hearts were harvested at 3, 7, and 14 days after surgery for different tests. **C** Echocardiographic measurements of LVEF, LVEDD, and left ventricle end-systolic diameter (LVESD) in wild-type (WT) and Cre/TDT mice. *N* = 7 mice per group. Data were shown as mean ± SEM, ns, not significant, by Student’s *t* test. **D** Representative cross sections of Cre/TDT ventricles stained with tdTomato and CD34. **E** Representative flow cytometry analyses of freshly isolated bone marrow cells from Cre/TDT mice stained with tdTomato and APC-conjugated CD34 antibody. **F** Representative cross sections of Cre/TDT ventricles stained with tdTomato and CD31. **G** Representative cross sections of Cre/TDT ventricles stained with tdTomato and CD45. **H** Representative cross sections of Cre/TDT kidney and spleen tissue stained with tdTomato and CD34. **I** Representative tdTomato expression on cross sections of Cre/TDT ventricles from 1 week and 2 weeks post-I/R in border zones and remote zones.** J** Representative cross sections of Cre/TDT ventricles stained with Ki67 and EdU. Magnification in each image was the magnification of the boxed region, arrows indicated co-staining cells. Scale bars, 100 μm, and 20 μm in magnification images. *tdT* tdTomato

A *Cd34*-CreER^T2^; R26-tdTomato lineage-tracing mouse model (Cre/TDT, the *Cd34*-lineage cells were permanently labeled with tdTomato upon tamoxifen administration) was developed and subjected to I/R surgery and single-cell RNA sequencing (scRNA-seq, Fig. 1A) to fully explore the roles of CD34^+^ cells in vivo. We performed echocardiography imaging as a noninvasive method to evaluate cardiac physiology in knock-in animals [19]. Infarction was established by echocardiogram-measured reduced cardiac function and Masson’s trichrome–stained fibrosis (Fig. S1). All the indexes, including left ventricular ejection fraction, left ventricular end-systolic diameter, and left ventricular end-diastolic diameter, exhibited similar results between Cre/TDT and wild-type C57BL6/J animals (Fig. 1B, C). Successful labeling of *Cd34*-lineage tdTomato (tdT)^+^ cells was observed in the heart and major organs, with partially co-expressed endothelial cell (EC) marker CD31 but seldom co-stained with leukocyte marker CD45 (Fig. 1D–H). The accumulation of tdT^+^ cells was observed in the border zones of the infarcted area, which indicated the involvement of CD34^+^ cells in tissue repair after I/R injury (Fig. 1I). Meanwhile, tdT^+^ cells co-stained with Ki67 and EdU were also observed, indicating their proliferating state (Fig. 1J).

**Fig. 2:**
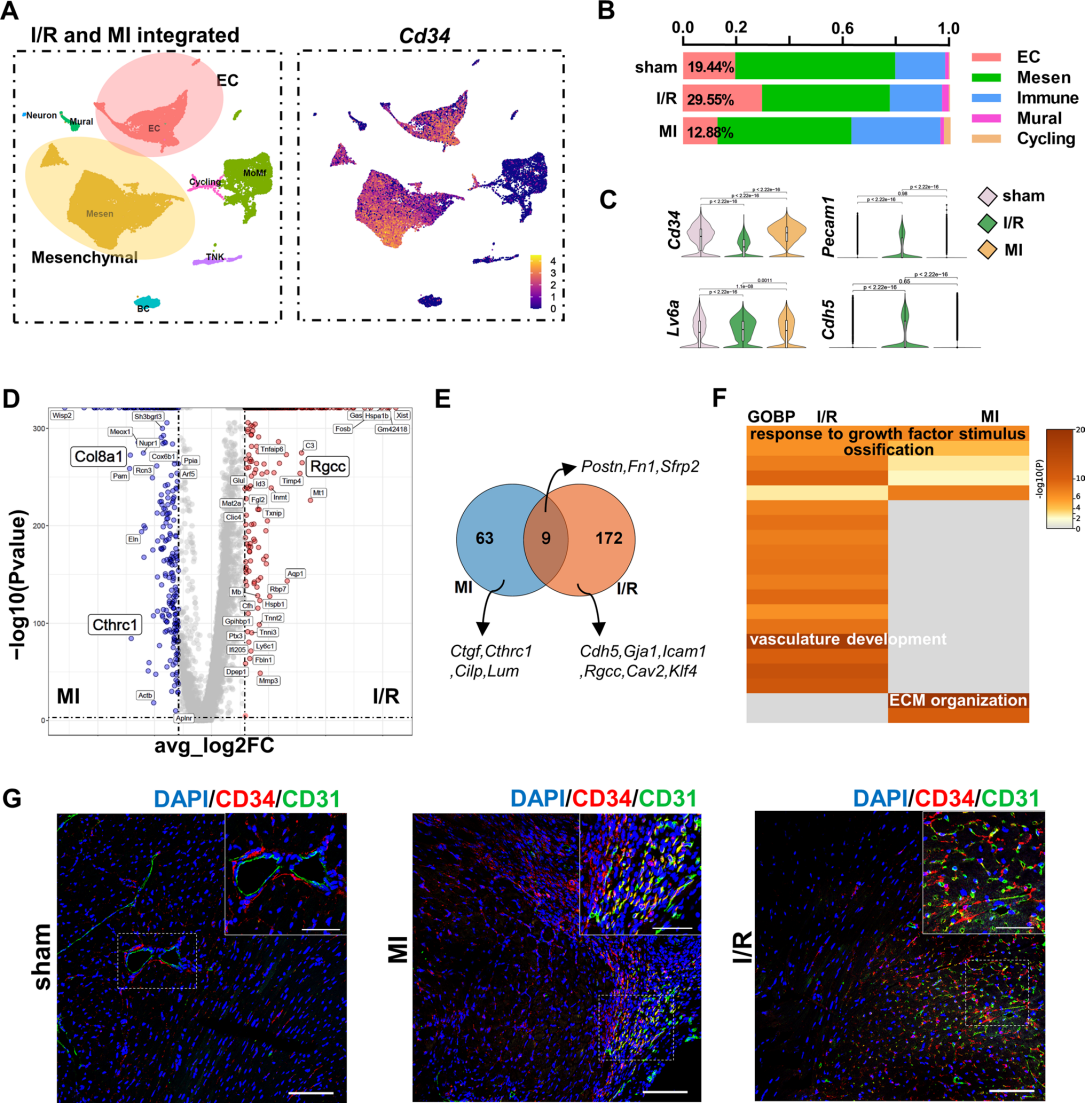
ScRNA-seq of non-CM cardiac cells revealed the contribution of *Cd34*-lineage cells in ischemia/reperfusion (I/R) injury. **A** Integration analysis of our total-cell I/R injury datasets, and one published myocardial infarction (MI) injury scRNA-seq datasets (datasets at the same time points with I/R in E-MTAB-7895) was performed to compare mouse I/R and MI models. Uniform Manifold Approximation and Projection (UMAP) visualization of eight color-coded major cell types, *n* = 29,407 cells. Feature plot on the right showed the expression of gene *Cd34* across cell types. **B** Bar chart showing the proportion of major cell types among different diseases in the integrated scRNA-seq dataset. **C** Violin plots displaying the gene expression levels of progenitor-like markers *Cd34, Ly6a* and endothelial cell (EC) markers *Pecam1* and *Cdh5* in mesenchymal (Mesen) and EC populations among different disease models. Statistical *p* values were added between each two groups. *P* < 0.05 was considered significant. **D** Volcano plot showing gene features between *Cd34*-expressing mesenchymal/ECs from the I/R dataset and MI dataset. **E** Venn plot exhibiting the intersection of upregulated genes in Mesen and EC populations of I/R or MI injury when compared to the sham group, respectively. Representative unique genes in each disease model and shared genes were labeled. **F** Go enrichment biological process (GOBP) analysis of unique genes in different diseased models revealed in (**C**), with putative major biological functions showed. **G** Representative cross sections of mouse ventricles from different disease models stained with CD34 and CD31 in border zones, with magnification of the boxed region. Scale bars, 100 μm, and 20 μm in magnification. CM, cardiomyocyte

The infarcted hearts were harvested at sham, 1 and 2 weeks after the I/R surgery, fully digested, and applied to scRNA-seq to illustrate the cellular changes after the I/R injury at the single-cell level. A median detection of 2000–2500 genes per cell was established by scRNA-seq (Fig. S2). We first combined the total-cell datasets with published murine MI datasets [6] to compare the differences in the two disease models. *Cd34* was mainly expressed in mesenchymal cell and EC populations in both models (Fig. 2A). Interestingly, the I/R model possessed unique cell components and gene profile so that a large number of ECs were preserved compared with those in either the sham or MI group (Fig. 2B). The mesenchymal cells and ECs, as the dominant and *Cd34*-expressing cell types in heart, showed decreased expression of progenitor genes *Cd34 and Ly6a* (stem cells antigen-1, Sca-1) but higher expression of EC markers (*Pecam1* encoding CD31 and *Cdh5* encoding vascular endothelial–cadherin) in the I/R injury (Fig. 2C). Referring to the upregulated genes or comparative DEGs in mesenchymal cells and ECs from I/R or MI datasets (Fig. 2D, E), profibrogenic genes were either seldom shared [Periostin (*Postn*)*, Fn1,* and *Sfrp2*] between models or largely enriched in MI (*Ctgf, Cthrc1, Cilp, Lum,* and *Col8a1*). However, the angiogenic genes (*Cdh5, Gja1, Rgcc, Icam1, Cav2,* and *Klf4*) were principally enriched in the I/R dataset, leading to a unique biological process (BP) of vasculature development predicted in the I/R injury (Fig. 2F), in which *Cd34*-lineage cells might actively take part. The following immunostaining on tissue sections from both diseases also confirmed the participation of CD34^+^ cells in angiogenesis after cardiac injury (Fig. 2G). As *Cd34* was mainly expressed on mesenchymal cells, we further compared our dataset with canonical *Pdgfra*-lineage cardiac mesenchymal cells using a public dataset from a *Pdgfra*^GFP/+^ mouse line to extensively address the biological properties of *Cd34*^+^ cell [5]. *Cd34* was exclusively expressed in ECs and a minor population of immune cells compared with the *Pdgfra*^+^ cells, while *Pdgfra*^+^ cells were almost only mesenchymal cells (Fig. S3A and S3B). The *Cd34*-lineage cells also exhibited exclusive capacities of vasculature development and cell differentiation (Fig. S3C and Table S1).

### Active profibrogenic capacity of *Cd34*-lineage mesenchymal cells revealed by scRNA-seq

The infarcted ventricles were harvested and digested at indicated time points, sorted based on the tdT expression, and applied to scRNA-seq to illustrate the dynamics of *Cd34*-lineage cells after the I/R injury (Fig. S4A). A total of 26,320 single cells from 6 datasets (total/tdT^−^ cells and sorted tdT^+^ cells from 3 time points) were integrated, and 7 major cell types were identified (Fig. S4B). The *Cd34*-lineage tdT^+^ cells were wildly distributed in mesenchymal cells, ECs, and monocyte/macrophage populations. Previous studies confirmed the role of bone marrow-originated *Cd34*-derived myeloid cells in cardiovascular diseases, which is a distinct developmental origin from their tissue-resident mesenchymal cells and EC counterparts [13].

**Fig. 3:**
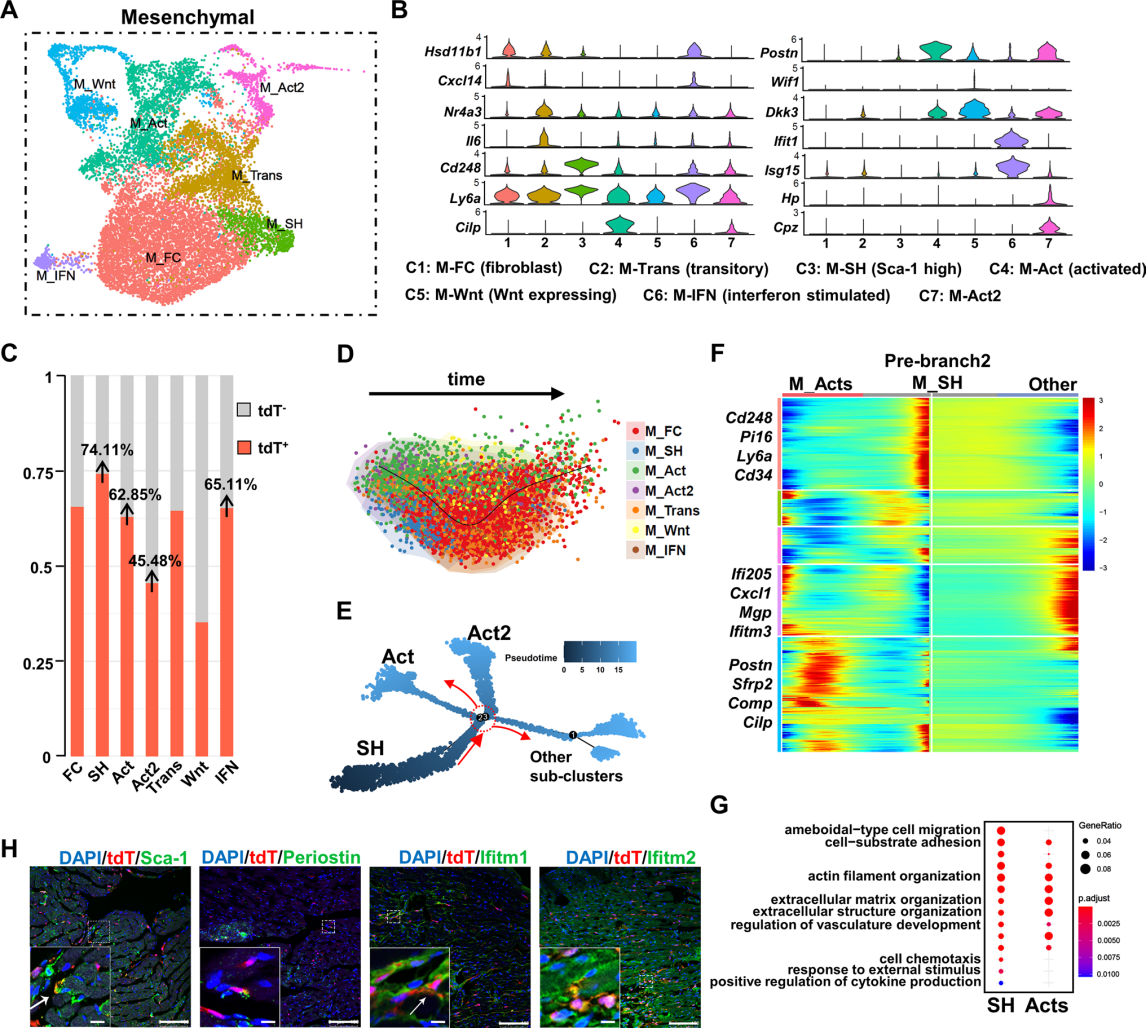
Contribution of *Cd34*-lineage mesenchymal cells in I/R injury. **A** UMAP plot showing 7 color-coded subclusters from the mesenchymal (Mesen) cell population in I/R injury, *n* = 14,125 cells. **B** Total-cell and tdTomato^+^ (tdT^+^) cell datasets from all time points (sham, I/R 1w, I/R 2w) were integrated, mesenchymal cell population were subclustered for deep investigation. Violin plot showing expression of selected marker genes across all mesenchymal cell subclusters, with putative cell identities annotated below. **C** Proportion of tdT^+^ cells in each mesenchymal cell subcluster. **D**, **E** SCORPIUS **(D)** and Monocle **(E)** trajectory analyses showing the relationship among tdTomato^+^ mesenchymal cell subclusters and potential differentiation directions over pseudotime. The branch point 2 selected for downstream analyses is indicated by the red circle. **F** Heatmap of the significantly changed genes (*P* < 0.01) discovered by the BEAM function from monocle in the branch point 2 in **(E)**, with selected upregulated genes listed at different cell states (prebranch, cell fate 1 and 2). **G** GOBP analysis of significantly changed genes (*P* < 0.01) in different cell fates discovered by the BEAM function from Monocle in the first branch point (branch point 2) from the trajectory in **(E)**. **H** Representative images of Cre/TDT ventricles staining tdT with Sca-1, Periostin, Ifitm1 and Ifitm2 in border zones, with magnification of the boxed region, arrows indicated co-staining cells. Scale bars, 100 μm, and 20 μm in magnification. IFN, interferon stimulated. Mesen, mesenchymal cell. *tdT* tdTomato

The I/R injury activated the mesenchymal cells to produce extracellular matrix (ECM), which formed a long-term persistent fibrotic scar in the wounded area [16]. As a major component of *Cd34*-lineage cells, a total of 14,125 mesenchymal cells were divided into 7 finer subclusters by focused clustering analysis (Fig. 3A and Table S2). The number of *Postn*-expressing activated mesenchymal cell subclusters (M_Act/Act2) significantly increased after the injury (Fig. 3B and Fig. S5A and S5B), which were believed to be major contributors to cardiac fibrosis [5]. Although tdT^+^ mesenchymal cells shared similar dynamic changes to tdT^−^ ones (Fig. S5B), the tdT^+^ cells mostly consisted of disease-responding mesenchymal cell subclusters, including M_Act/Act2, interferon-stimulated mesenchymal cell (M_IFN), and especially Sca-1 highly expressing ones (M_SH, 74.11%), rather than the Wnt-expressing subcluster (M_Wnt), which was anti-fibrotic (Fig. 3C) [5]. Pseudotime trajectory analyses using SCORPIUS and Monocle methods exhibited an initiative cell state of M_SH and a potential differentiation process toward M_Act/Act2 (Fig. 3D, E and Fig. S5C). The upregulated genes at the branch point where M_SH differentiated into M_Act/Act2 (Fig. 3F) revealed distinct cellular functions. The M_SH was characterized to be involved in BPs, including cell migration, cell adhesion, active response to stimuli or cytokines/chemokines, and regulation of vasculature development. However, the M_Acts clusters showed a pro-fibrotic phenomenon such as ECM organization (Fig. 3G and Table S1). We also evaluated our findings in vivo through immunostaining on tissue sections. Noticeable tdT^+^ cell-involved cardiac fibrosis was observed after I/R injury according to vimentin and collagen I staining (Fig. S5D), and tdT^+^ M_SH/M_Acts/M_IFN were also identified (Fig. 3H) with evident co-expression of tdT and Sca-1, *Postn* encoded, and Ifitm1/2. Of note, according to the aforementioned data, *Cd34*-lineage mesenchymal cells dominated the M_SH subtype (Fig. 3C). However, the initial active player M_SH, co-expressing *Ackr3*, *Cd34*, *Cd248*, and *Pi16* (Fig. 3B, F), was reported as cardiac mesenchymal-like stem cells with multilineage differentiation and self-renewal capacities, and promoted heart failure [2, 5]. Unlike most classic myofibroblasts, which normally faded within 2 weeks [8], the M_Acts subclusters (*Postn*^+^, *Acta2*^low^), which were also tdT^+^ cells largely constituted subtypes, kept increasing until 2 weeks after I/R injury and expressed *Cilp* (encoding CILP1), indicating a pro-fibrotic phenotype crucial in cardiac remodeling and fibrosis [21]. The other tdT^+^ cell highly contributed to the M-IFN subcluster, which, despite comprising a minor population and remaining stable over time, might slow down the progression of cardiac fibrosis [15]. Collectively, *Cd34*-lineage mesenchymal cells played dominant roles in post-I/R cardiac fibrosis.

### Angiogenic property of *Cd34*-lineage ECs in cardiac I/R injury

**Fig. 4:**
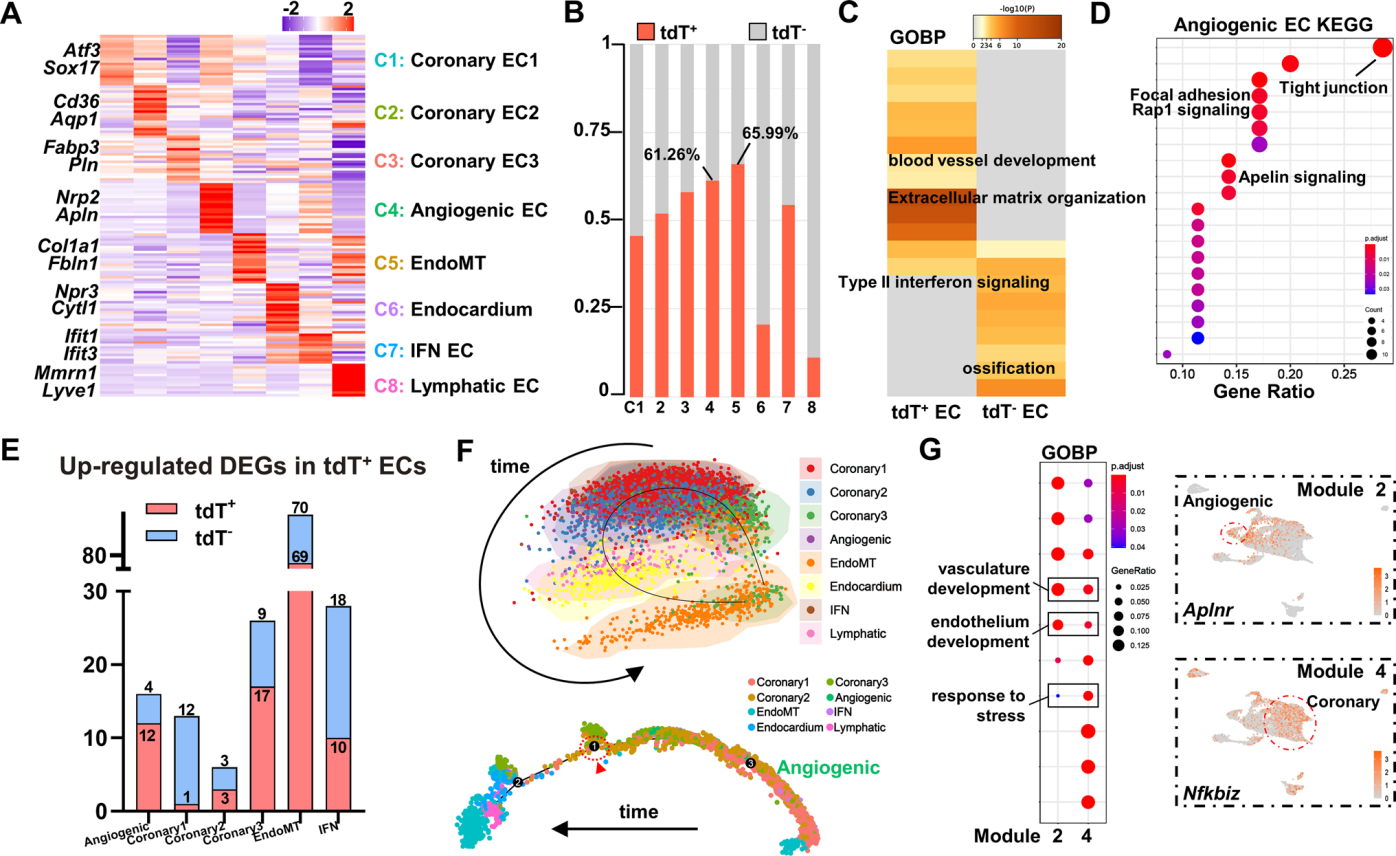
Contribution of *Cd34*-lineage endothelial cells in I/R injury. **A** Heatmap showing average scaled expression levels of top 20 differentially expressed genes (DEGs) across EC subclusters, with representative 2 markers listed to help define putative biological identities listed on the left. **B** Proportion of tdT^+^ cells in each EC subcluster. **C** GOBP analysis of DEGs between tdT^+^ ECs and tdT^−^ ECs showing different biological functions. **D** Kyoto Encyclopedia of Genes and Genomes (KEGG) analysis of the angiogenic EC cluster using marker genes to show putative functions and pathways. **E** Numbers of *Cd34*-lineage-related DEGs (tdT^+^ and tdT^−^) in the EC subsets. **F** SCORPIUS (upper) and Monocle (below) trajectory analyses showing cell distribution over pseudotime, colored by cell clusters. The branch point 1 selected for further analyses was indicated by the red circle and arrow head. **G** GOBP analysis of significant genes relating to the two prebranch gene modules (Module 2 and 4) via BEAM function at Monocle EC trajectory branch point 1, with feature plots showing representative genes *Aplnr* and *Nfkbiz* from each module. EndoMT, endothelial-to-mesenchymal transition. IFN, interferon stimulated. *tdT* tdTomato

We also performed high-resolution analyses on the other main components of tissue-resident *Cd34*-lineage cells, the cardiac ECs. The ECs were further characterized into eight subclusters according to their gene profile and putative biological functions (Fig. 4A and Fig. S6A–S6D and Tables S3 and S4). The angiogenic EC subcluster, highly expressing tip/stalk cell markers (*Nrp2*, *Apln*), and the ECM gene-expressing endothelial-to-mesenchymal transition (EndoMT) subcluster were two major tdT^+^ cell-contributing cell types (over 60%; Fig. 4B). Comparative DEG analyses between tdT^+^ and tdT^−^ ECs revealed specifically enriched BPs, including blood vessel development and ECM organization in tdT^+^ ECs (Fig. 4C and Fig. S6E and Table S1). As the subtype that tdT^+^ cells contributed the most, the angiogenic EC possessed higher abilities of angiogenesis, cell junction organization, and focal adhesion, with several canonical angiogenesis-related pathways (Rap1 signaling and Apelin signaling) enriched (Fig. 4D and Fig. S6F). Besides, tdT^+^ angiogenic ECs exhibited amplified DEGs compared with their tdT^−^ counterparts, indicating a more active cellular response to I/R injury (Fig. 4E). Pseudotime trajectory analyses also showed an initial cell state and differentiating potential from angiogenic ECs toward other cell subtypes (Fig. 4F and Fig. S6G). Although we found that the angiogenic ECs might be the source of postinjury angiogenesis, the coronary ECs were also distributed at the primitive cell state on the trajectory. DEGs were acquired according to different gene profiles at the branch point 1 to delineate the true source of angiogenesis, and two distinct gene modules were identified in the prebranch primitive state (Fig. S6H). Combining BP and pathway enrichment analyses, gene module 2 was found to be related to angiogenesis by its enriched vasculature/endothelium development BPs and pathways, while gene module 4 was more like a pattern relevant to disease response (Fig. 4G and Fig. S6I and Table S1). Additional examination of DEGs in the two modules further revealed that module 2 (*Aplnr*) represented angiogenic ECs while module 4 (*Nfkbiz*) was a representative to coronary ECs, which strongly indicated that *Cd34*-lineage angiogenic ECs were the actual source of post-I/R angiogenesis (Fig. 4G and Fig. S6J).

### Pro-inflammatory features of circulating *Cd34*-lineage myeloid cells

**Fig. 5:**
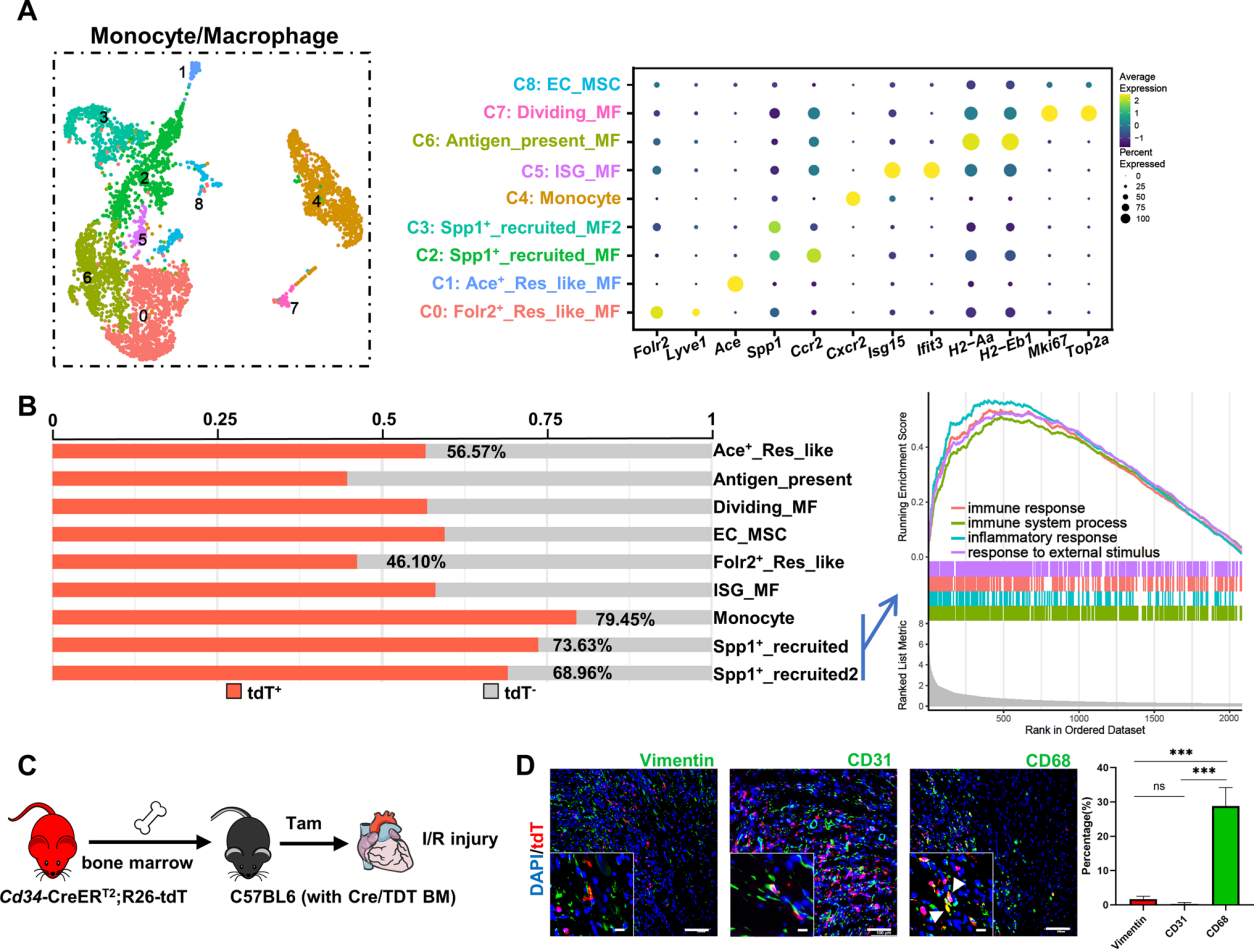
Contribution of *Cd34*-lineage monocytes/macrophages in I/R injury. **A** UMAP plot showing 9 color-coded subclusters from the monocytes/macrophages population in I/R injury, *n* = 4579 cells. Dot plot shown on the right indicating the expression of selected marker genes across all subclusters, with putative cell identities annotated in the middle. **B** Proportion of tdT^+^ cells in each monocyte/macrophage subcluster. Gene set enrichment analysis (GSEA) shown on the right indicated significant pathways enriched in recruited subclusters (monocytes, Spp1^+^ macrophages). **C** Sketch of the experimental design for generating chimeric C57BL6 mice with bone marrow cells transplanted from Cre/TDT mice. Chimeric mice were subjected for I/R injury and heart samples were harvested for further investigation. **D** Representative images of chimeric C57BL6 murine ventricles staining tdT with vimentin, CD31 and CD68 in border zones, with magnification of the boxed region, arrows indicated co-staining cells. Scale bars, 100 μm, and 20 μm in magnification. Quantification of the percentages of vimentin^+^/CD31^+^/CD68^+^ cells in BM-derived tdTomato^+^ cells by immunofluorescence was shown on the right panel. *N* = 4 mice, ***P < 0.001, by one-way ANNOVA test. *BM* bone marrow, *ISG* interferon stimulated, *tdT* tdTomato

After cardiac I/R injury, both resident and circulating monocytes/macrophages infiltrated into the injured area to exert heterogenous effects such as modulating inflammation response, engulfing cell debris, regenerating cardiac tissues, and initiating fibrotic scar formation [4]. Nine finer subclusters of monocytes/macrophages were identified in our scRNA-seq datasets, with distinct biological functions predicted according to their gene profiles (Fig. 5A and Fig. S7A, and Table S5). The monocytes and *Spp1*^+^ subclusters were classified as recruited circulating myeloid cells via the expression of canonical *Spp1*, *Ccr2*, and *Cxcr2* markers. Another two *Folr2/Lyve1/Ace*-expressing subclusters were identified as resident-like macrophages. Other minor populations included interferon-stimulated gene (ISG) macrophages (*Isg15* and *Ifit3*), antigen-presenting macrophages (*H2-Aa* and *H2-Eb1*), and dividing macrophages (*Mki67* and *Top2a*). Comparing the percentage of tdT^+^ cells in each subcluster, the recruited monocytes/*Spp1*^+^ macrophages dominated the contribution of *Cd34*-lineage myeloid cells, with enriched cellular function of classical response to external stimulus, which was believed to be a trigger to postinjury inflammation (Fig. 5B). We next sought to specify the cellular characteristics of the macrophage system by assessing a previously established classification of classically activated (M1) and alternatively activated (M2) macrophages [24]. Major recruited macrophages were more like M1 macrophages, while resident macrophage subclusters acted as M2 macrophages, supporting their pro-inflammatory or anti-inflammatory properties, respectively. The antigen-presenting properties were seen mostly in the antigen-presenting subcluster and slightly in ISG macrophages, *Ace*^+^ resident-like macrophages, and dividing macrophages (Fig. S7B). Further enrichment analyses based on DEGs in each subcluster revealed a pro-inflammatory response of recruited macrophages and anti-inflammatory property of resident-like ones (Fig. 5B and Fig. S7C and S7D). The accumulation of abundant CD68^+^ tdT^+^ macrophages in the heart chamber in response to I/R injury was also validated in vivo (Fig. S7E).

As mentioned earlier, we previously confirmed a bone marrow origin of *Cd34*-lineage myeloid cells in a pathological vessel disease [13]. A bone marrow transplantation mode was performed and chimeric C57BL6 mice with Cre/TDT bone marrow cells were generated to address whether bone marrow-originated *Cd34*-lineage cells also participated in the I/R injury. Through tamoxifen administration, only bone marrow-originated *Cd34*-lineage cells could be labeled tdT expression in such chimeric mice (Fig. 5C). After 3 days post-I/R injury, a large number of tdT^+^ CD68^+^ macrophages were found in the chimeric heart sections, while the tissue-resident components (vimentin^+^ mesenchymal cells and CD31^+^ ECs) were rarely seen, which validated a bone marrow source of *Cd34*-lineage myeloid cells in vivo (Fig. 5D).

### *Cd34*-lineage cell-mediated active angiogenesis in I/R injury

**Fig. 6:**
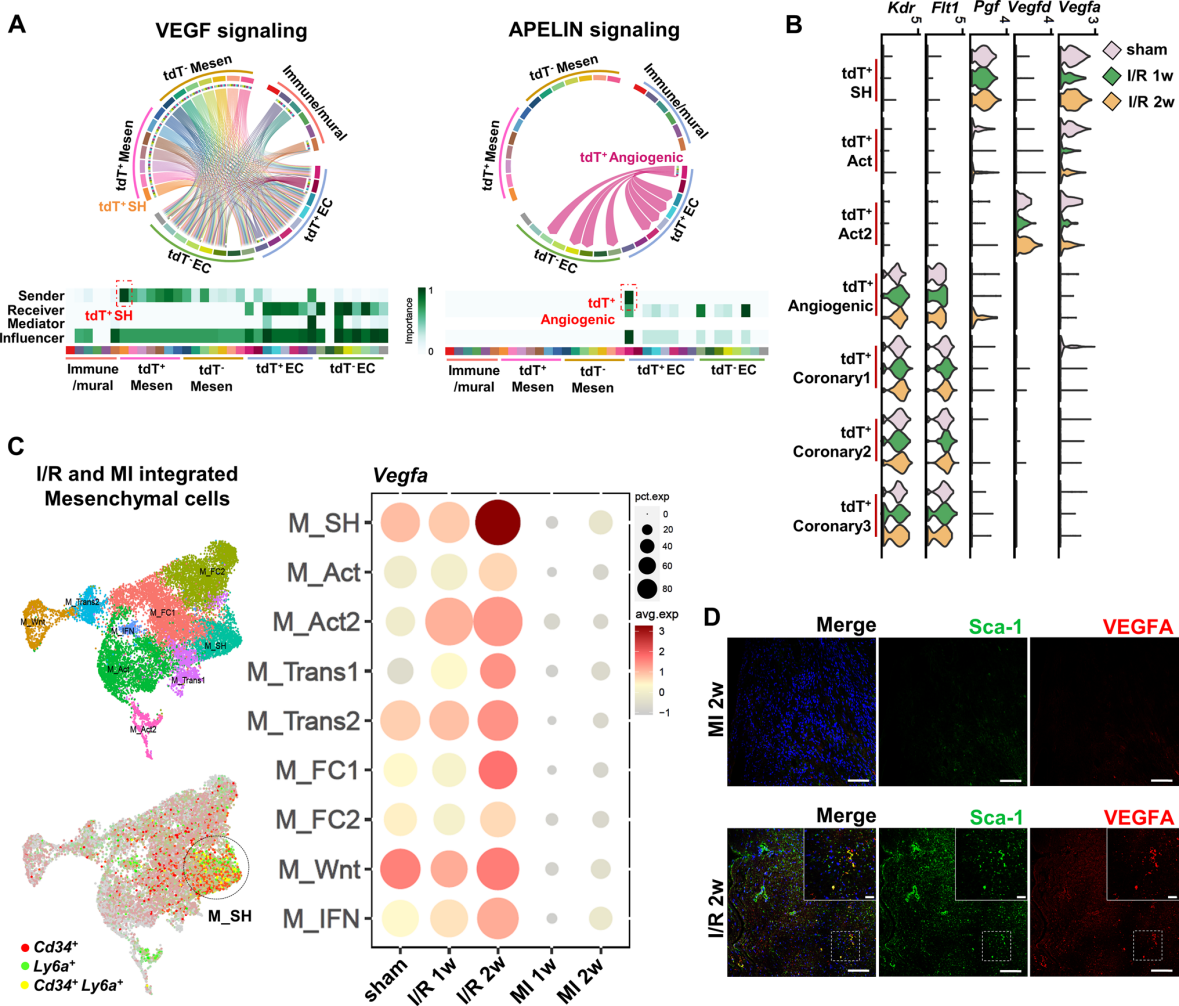
Pathological impact of *Cd34*-lineage cells on ischemic heart. **A** Chord plots showing dominant VEGF signaling between mesenchymal and EC populations and angiogenic ECs’ specific APELIN signaling by CellChat intercellular communication analyses. Heatmaps showing the relative importance of each cell group based on the computed four network centrality measures of each signaling network. **B** Violin plots displaying expression profile of major VEGF signaling ligand genes (*Vegfa*, *Vegfd*, *Pgf*) in tdT^+^ SH/ Act/ Act2 clusters and relevant receptor genes (*Flt1*, *Kdr*) in tdT^+^ angiogenic/coronary ECs among different time points after I/R injury. **C** Umap clustering of mesenchymal cells from integrated our total-cell I/R and public MI datasets [7] (left panel), with *Cd34*^+^, *Ly6a*^+^ and *Cd34*^+^ *Ly6a*^+^ cells identified. Dot plot showing *Vegfa* expression across all mesenchymal subclusters among different I/R or MI time points. **D** Representative images of ventricles harvested from C57BL6 mice subjected to different surgeries (I/R or MI) staining Sca-1 and VEGFA, with magnification of the boxed region. Scale bars, 100 μm, and 20 μm in magnification. *EC* endothelial cell, *Mesen* mesenchymal cell, *tdT* tdTomato, *I/R* ischemia/reperfusion, *MI* myocardial infarction

The existence of intercellular interaction among cardiac cells was addressed. The mesenchymal cells were able to promote angiogenesis [33], and ECs had an impact on the phenotypic switch of mesenchymal cells [17]. We first assessed the proliferating property of *Cd34*-lineage cells and found a high proportion of EdU^+^tdT^+^ cells in all mesenchymal cells, ECs, and macrophages, which indicated their active participation after the I/R injury (Fig. S8). We next aimed to decipher intercellular communicating patterns between these two cell populations, especially in *Cd34*-lineage tdT^+^ cells. A thorough evaluation of cellular communicating networks revealed that the vascular endothelial growth factor (VEGF) and SEMA3 signaling, with the Vegfa-Vegfr1/2 and Sema3c-Nrp1/2 ligand–receptor pairs, were found to be the major influencers in M_SH-angiogenic EC interaction. The EndoMT cells mainly received the Gas6 signal via their Axl receptors from M_Acts. Angiogenic and coronary ECs, in turn, communicated with M_SH cells via CXCL (Cxcl12) and MIF (Mif) signaling, while Ackr3 expressed on M_SH played a crucial role in receiving signals from ECs (Fig. S9A). Especially, tdT^+^ M_SH was the most active pro-angiogenesis VEGF signal sender to take effect on ECs, while tdT^+^ angiogenic ECs obtained unique Apelin signaling to interact with other EC subtypes (Fig. 6A). Further analyses also revealed I/R injury-related signaling (Fig. S9B), such as fibrosis-related pleiotrophin, periostin signaling, and inflammation-related SPP1 signaling in the I/R 1w (1 week after I/R injury) dataset, in which M_Acts vigorously took part. In the I/R 2w (2 weeks after I/R injury) group, the noncanonical WNT signaling regulated the cross talk between M_SH and M_Acts, and a late-phase angiogenic response was found to be enhanced by tdT^+^ angiogenic ECs via Apelin signaling (Fig. S9C and S9D).

The M_SH cells, especially the *Cd34*-lineage tdT^+^ ones, exhibited the highest and strongest cell–cell communicating activity across all cell types (Fig. S9E). Of note, unlike traditional concepts that inflammation induces postinjury angiogenesis, our findings of intercellular communication revealed that tdT^+^ mesenchymal cells (especially tdT^+^ M_SH) dominated a pro-angiogenesis effect on ECs compared with immune cell types. Interestingly, the capacity of *Vegfa* expression in tdT^+^ M_SH, despite displaying a decrease 1 week after I/R, restored in the second week (Fig. 6B and Fig. S9F). Compared with the MI dataset, M_SH exhibited unique *Vegfa* expression, the highest in the second-week I/R group (Fig. 6C, D), which might explain the higher percentage of EC reserved in I/R than MI. In summary, *Cd34*-lineage tdT^+^ M_SH and angiogenic ECs led the cross talk among cardiac cells.

### Inducible ablation delineated the role of *Cd34*-lineage cells in cardiac fibrosis

**Fig. 7:**
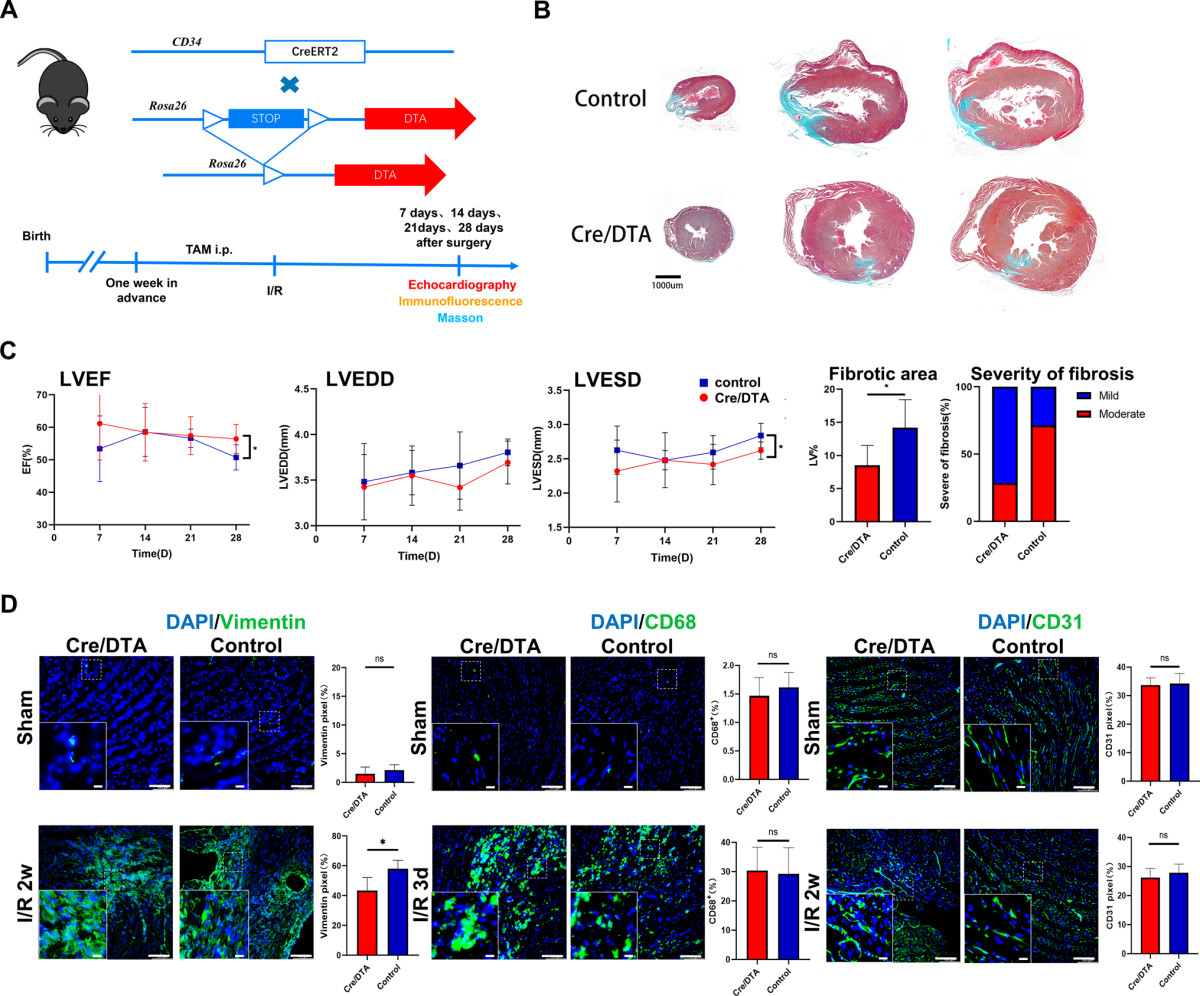
Pathological impact of *Cd34*-lineage cells on ischemic heart. **A** Experimental scheme whereby *Cd34*-CreER^T2^;R26-DTA (Cre/DTA) or control mice were given tamoxifen for 1 weeks before I/R injury. Hearts were harvested at 7, 14, 21, and 28 days after surgery. **B** Masson staining showing cross sections of different horizontal planes (apex, left ventricles) of hearts from Cre/DTA or control mice at 28 days post-I/R, indicating different degrees of fibrosis. Scale bar, 1 mm. **C** Echocardiographic measurements of left ventricle ejection fraction (LVEF), left ventricle end-diastolic diameter (LVEDD) and left ventricle end-systolic diameter (LVESD) in Cre/DTA and control mice at the indicated time after I/R injury, and percentage of fibrotic area (area of scar, normalized to the area of the ventricle) along with severity of fibrosis (fraction of mice demonstrating mild or moderate scarring) after 28 days post-I/R in two mouse groups. *N* = 7 mice per group. **D** Representative images of Cre/DTA or control ventricles staining vimentin, CD68 or CD31 showing cellular changes of mesenchymal cells, macrophages or endothelial cells after CD34^+^ cell ablation, with magnification of the boxed region. *N* = 5 mice per group. Scale bars, 100 μm, and 20 μm in magnification. Data were shown as mean ± SEM, *P < 0.05, by one-way ANNOVA test or Student’s *t* test

We next sought to find out how *Cd34*-lineage cells were involved in I/R injury in vivo. A *Cd34*-CreER^T2^; R26-eGFP-DTA (Cre/DTA) mouse line was generated to conduct a tamoxifen-induced diphtheria toxin (DT)-mediated specific cell depletion of *Cd34*-lineage cells (Fig. S10A). No difference in cardiac function was found in Cre/DTA mice compared with the wild-type mice under normal conditions. The cell ablation was evaluated by immunostaining and flow cytometric analyses on both cardiac and bone marrow cells. Fewer CD34^+^ mesenchymal or EC were also found on heart tissue sections (Fig. S10B–S10E). The Cre/DTA mice were then subjected to I/R injury (Fig. 7A), and we surprisingly observed a recovered cardiac function and reduced fibrosis in Cre/DTA mice (Fig. 7B, C, and Fig. S10F). The cellular changes of cardiac cell components showed a significant decrease in mesenchymal cells, while no differences were found in macrophages or ECs between control and Cre/DTA mice (Fig. 7D). According to the aforementioned findings, this improvement in the cardiac function could be attributed to the annihilation of *Cd34*-lineage cell-conducted cardiac fibrosis. Unlike most clinical trials on accumulating circulating CD34^+^ cells, the intervention using ablating tissue-resident CD34^+^ cells exhibited a beneficial therapeutic effect in treating cardiac ischemic disease.

### *Cd34*-lineage cells contributed to pathological changes in the human ischemic heart

**Fig. 8:**
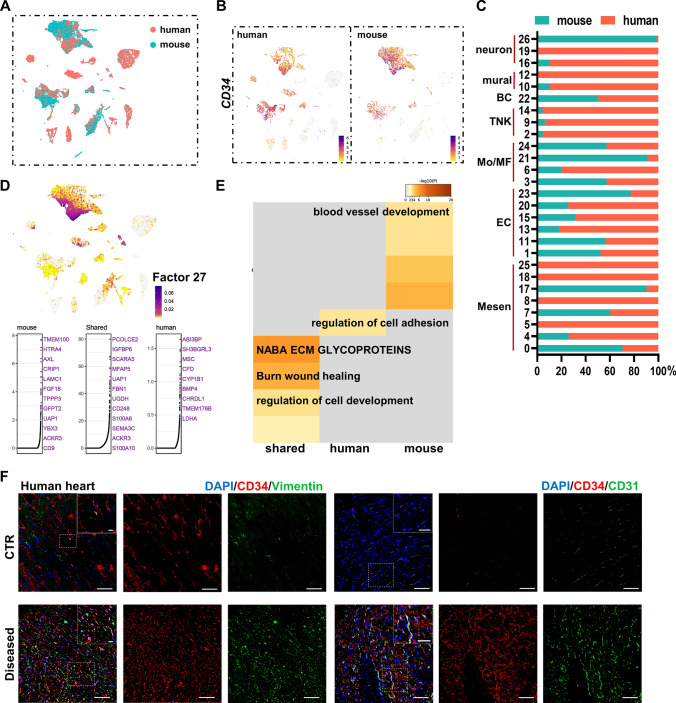
Validation of CD34^+^ cells in human heart. **A** UMAP visualization of human and mouse single cells analyzed by LIGER, color coded by species. **B** Feature plots exhibiting the expression of *CD34* across species. **C** Proportion of human and mouse cells in each single-cell cluster in the integrated dataset. **D** Cell factor loading values (upper) and gene loading plots (below) of left loading species-specific and shared genes for factor 27. **E** GOBP analysis of loaded genes in **(D)** indicating certain biological functions. **F** Representative images of CD34, co-staining with mesenchymal cell marker vimentin and endothelial cell marker CD31, in control and ischemic human hearts, with magnification of the boxed region. Scale bars: 100 μm, and 20 μm in magnification images. *BC* B cell, *TNK* T and natural killer (NK) cells, *Mo* monocyte, *MF* macrophage, *EC* endothelial cell, *Mesen* mesenchymal cell

Finally, we collected healthy and ischemic human cardiac samples for scRNA-seq from patients undergoing heart transplantation (Fig. S11A and Table S6) to investigate the evolutionary similarities and variations in gene expression profiles between human and mouse hearts. We compared human and mouse total-cell datasets using the LIGER algorithm (Fig. 8A). *CD34* was restrictively expressed in a mesenchymal cell and EC populations despite some cell-cluster variations between species (Fig. 8B, C and Fig. S11B), which was validated and also found to be enhanced in ischemia through immunostaining on cardiac tissue sections (Fig. 8F). Significant shared and dataset-specific gene markers were further recognized by the LIGER joint cluster analyses. A gene set in factor 27 showed high expression in cells with classical *CD34* expression pattern. Species-shared and species-specific genes of factor 27 were illustrated. The shared expression of *CD248*, *SEMA3C*, and *ACKR3* indicated a conserved existence of *Cd34*-lineage M_SH cells (Fig. 8D). Enriched analysis using genes from factor 27 showed shared BPs of wound healing and cell development, while vessel development was more active in mouse dataset and cell adhesion and found to be more enriched in human dataset (Fig. 8E).

## Discussion

After the I/R injury, the infarcted heart mainly undergoes inflammatory, reparative, and maturation phases, featuring inflammatory cell infiltration, tissue restoration, and scar maturation, respectively [7, 11, 25]. Both appropriate inflammatory activation and prompt restoration initiation are pivotal in cardiac wound healing [14, 27]. Since the isolation of CD34^+^ “putative EPCs” from peripheral blood mononuclear cells [1], many clinical studies reported that CD34^+^ cell therapy could treat cardiovascular diseases [20, 28]. However, the outcomes of these clinic trials varied tremendously [30, 32]. In the present study, we demonstrated that CD34^+^ cells mainly terminated into mesenchymal cells, ECs, and macrophages using lineage-tracing immunostaining and scRNA-seq techniques. We demonstrated that these diverse CD34^+^ cell-derived cells exerted their role by inducing inflammatory response, angiogenesis, and fibrosis in damaged tissues. Deleting CD34^+^ cells significantly improved the heart function and reduced scar formation. Thus, we provided the first evidence of the impact of endogenous CD34^+^ cells in cardiac remodeling.

The mesenchymal cells are the largest population derived from *Cd34*^+^ lineage cells, which are further divided into several subpopulations. The M-SH cluster was thought to comprise mesenchymal precursors to give rise to other types of mesenchymal cells by highly expressing *Ly6a* (Sca-1) and other classic progenitor markers. The pseudotime trajectory analysis also confirmed our findings that the M-SH cluster was located at the root of the trajectory and underwent a differentiation process along it. M-Act were thought to be the precursors of myofibroblasts [5]. This study showed that the percentage of M-Act kept increasing until 14 days after the cardiac injury, but the myomesenchymal cells normally faded within 2 weeks [8]. We therefore speculated that M-Act might represent an independent fibroblast population, with a similar cluster recently reported by Forte et al. [6] termed as late-resolution mesenchymal cells. In fact, this study identified that M-Act mainly expressed genes associated with matrix deposition and stress response, indicating their persisting role in accelerating fibrosis until the scar maturation stage after cardiac I/R injury. They can be deleterious to the heart function with unrestricted proliferation in the late stage by promoting unhalted inflammatory reaction, although fibrosis may play a role in maintaining the cardiac stability early after the MI.

Within the heart chamber after the I/R injury, the damaged tissue secretes pro-inflammatory cytokines to recruit both circulating and resident macrophages to remove injured tissue and be involved in cardiac remodeling. Disturbance of immune components leads to further impairment after cardiac ischemic injury [10]. We found that most tdT^+^ myeloid subclusters were identified as recruited circulating macrophages with M1 phenotype, which promoted inflammation [26]. We confirmed our findings in vivo through bone marrow transplantation experiments and enforced our previous theory that the bone marrow source of CD34^+^ cells only contributed to immune cells. This finding could be a convincing explanation for the failure of several clinical trials that bone marrow-derived CD34^+^ cells exaggerated inflammatory response in injured hearts and enhance lesion formation.

This study demonstrated that CD34^+^ cell-derived ECs were mainly responsible for arterial ECs, but minimally contributed to endocardium generation, both under physiological state and after I/R injury. Coronary arteries acted as cardiac intramural vessels to mainly maintain heart perfusion and supply nutrient substances to the cardiomyocytes [9]. In cardiac I/R injury, ischemic coronary arterial ECs may become more dysfunctional when the blood flow is reperfused [3]. The angiogenic EC cluster, highly expressing EC cell markers (*Cd34, Apln,* and *Nrp2*), was enriched for the activated function of angiogenesis and was distributed at the root of a pseudotime trajectory, indicating an important source of endothelial regeneration after cardiac injury. After infarction, ECs may undergo plastic changes including metabolic alteration, mesenchymal transition, hematopoietic and pro-inflammatory responses, angiogenesis and proliferation [34]. Our results indicated that CD34^+^ cell-derived ECs exhibited active proliferative capacity, which may be beneficial to post-infarction angiogenesis and restoration of local microcirculation, thereby to some extent limited the infarct area.

By intercellular communication analysis, we identified several ligand-receptor pairs that dominated the cross talk within mesenchymal and EC populations. The tdT^+^ mesenchymal cells were the sole source of VEGF secretion, and tdT^+^ M_SH was the strongest player within them. Interestingly, the VEGF signaling mediated by tdT^+^ M_SH first decreased after 1 week of injury, but was soon restored after a 2-week time point, which might explain a more active angiogenesis and more EC restoration in I/R injury. We may further develop novel pro-angiogenesis treatment for ischemic heart disease by mediating VEGF secretion and targeting CD34^+^ M_SH cells.

Although the heterogenous progeny phenotypes of CD34^+^ cells, including mesenchymal cells, ECs, and macrophages, acted with diverse functions after the I/R injury, CD34^+^ cell ablation led to the net effect of a relief on fibrotic lesions 4 weeks after infarction. We speculated that CD34^+^ cell-derived M_SH and M-Act mesenchymal cells dominated fibrosis modulation during the maturation period. It should be noted that ECs’ abundance was not affected after CD34^+^ cell ablation according to our results of immunofluorescence. This might be due to discrepancies of Cre expression in different cell types, diverse efficiency of DT-induced cell ablation for different CD34^+^ subpopulations, or compensatory expansion of CD34^−^ ECs after ablation. The role of CD34^+^ ECs might be masked. Further studies using dual lineage-tracing systems to conduct more specific ablation of CD34^+^ ECs or CD34^+^ mesenchymal cells may provide insights into exact roles of each CD34^+^ subpopulation.

We confirmed a similar CD34^+^ cell distribution and cellular function by validating the findings using human scRNA-seq data, which indicated that our findings using mouse models could be further validated in humans.

In conclusion, our findings demonstrated the heterogeneity of tissue *Cd34*–derived cells in the heart that contributed to multilineage cell types and exhibited unique cellular functions in I/R injury. For example, *Cd34*-lineage tdT^+^ Acts led to fibrosis, tdT^+^ M_SH was an active player that regulated fibrosis and stimulated persistent angiogenesis, tdT^+^ angiogenic ECs were the main source of postinjury microcirculation restoration, bone marrow *Cd34*-lineage cell-derived circulating myeloid cells caused inflammation, and the depletion of tissue CD34^+^ cells relieved postinjury cardiac fibrosis. This study provided new insights that cardiac CD34^+^ cells might serve as therapeutic targets for the cardiac regeneration and improvement in heart function.

### Supplementary Information

Below is the link to the electronic supplementary material.

Supplementary file1 (XLSX 40 KB)

Supplementary file2 (XLSX 168 KB)

Supplementary file3 (XLSX 277 KB)

Supplementary file4 (XLSX 43 KB)

Supplementary file5 (XLSX 438 KB)

Supplementary file6 (XLSX 12 KB)

Supplementary file7 (PDF 11506 KB)

Supplementary file8 (DOCX 67 KB)

## Data Availability

The data and R scripts related to the findings of this study are available on reasonable request. The raw scRNA-seq data reported in this study have been deposited in the Genome Sequence Archive [35] in National Genomics Data Center [22], Beijing Institute of Genomics (China National Center for Bioinformation), and Chinese Academy of Sciences, under accession numbers CRA005739 and HRA001765, respectively, and are publicly accessible at https://ngdc.cncb.ac.cn. Two previously published scRNA-seq datasets used in this study are available in the ArrayExpress repository (E-MTAB-7895 [6] and E-MTAB-7376 [5]).
