# Supplementary material for: Multilineage contribution of CD34+ cells in cardiac remodeling after ischemia/reperfusion injury
Source: Basic Res Cardiol. 2023 May 5;118(1):17. doi: 10.1007/s00395-023-00981-8 (PMC10163140; doi:10.1007/s00395-023-00981-8)
Supplement: Supplementary file 7 — Supplementary file7 (PDF 11506 KB) [file 395_2023_981_MOESM7_ESM.pdf]

Supplementary Figures

Multilineage Contribution of CD34⁺ Cells in Cardiac Remodeling after Ischemia/Reperfusion Injury

Running title: $CD34^+$ Cells in Myocardial I/R Injury

Jun Xie [1†], Liujun Jiang [2†], Junzhuo Wang [1†], Yong Yin [1], Ruilin Wang [2], Ting Chen [2,3], Zhichao Ni [2], Shuaihua Qiao [1], Hui Gong [2], Biao Xu [1*], Qingbo Xu [2*]

[1]Department of Cardiology, Drum Tower Hospital, State Key Laboratory of Pharmaceutical Biotechnology, Medical School of Nanjing University, Nanjing, China;

[2]Department of Cardiology, the First Affiliated Hospital, Zhejiang University School of Medicine, Hangzhou, China;

[3]Alibaba-Zhejiang University Joint Research Center of Future Digital Healthcare, Hangzhou, China.

*Corresponding author. Biao Xu, MD, PhD, Department of Cardiology, Drum Tower Hospital, State Key Laboratory of Pharmaceutical Biotechnology, Medical School of Nanjing University, No. 321 Zhongshan Road, Nanjing, 210008, Jiangsu, China. Phone and Fax: +86 25-68182812, E-mail: xubiao62@nju.edu.cn. And Qingbo Xu, MD, PhD, Department of Cardiology, the First Affiliated Hospital, Zhejiang University School of Medicine, 79 Qingchun Road, Hangzhou, Zhejiang Province, P.R.China, 310003. Tel: +86 571-87236500, Fax: +86 571 4008306430, Email: qingbo_xu@zju.edu.cn.

[†] These authors contributed equally to this work.

Fig. S1

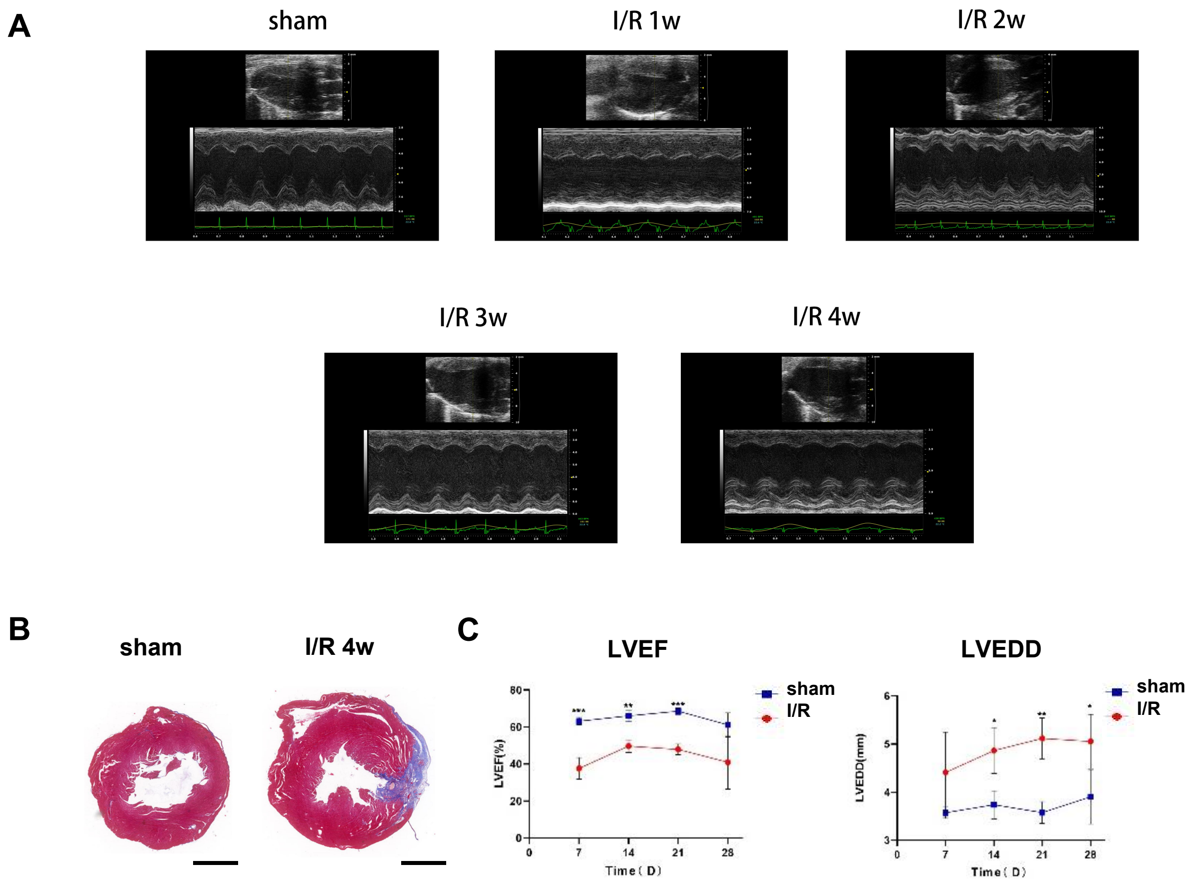

Figure S1. Establishment of ischemia/ reperfusion (I/R) injury model in mouse. (A) Representative echocardiogram of injured mice at different time points. **(B)** Masson staining of hearts sectioned at 28 days post-I/R. Scale bar, 1mm. **(C)** Line charts showing echocardiographic measurements of left ventricle ejection fraction (LVEF) and left ventricle end diastolic diameter (LVEDD) in mice from sham or different I/R groups. n=4 mice in I/R groups and n=3 in sham group. Data was shown as mean ± SEM, *p<0.05, **p<0.01, ***p<0.001, by one-way ANOVA test.

Fig. S2

A

PARAMETER	I/R sham	I/R 1w	I/R 2w	I/R sham tdT⁺	I/R 1w tdT⁺	I/R 2w tdT⁺	human control	human diseased
Estimated Number of Cells	4,138	6,020	6,259	5,661	6,919	6,567	16,635	10,148
Number of Reads	343,924,898	366,396,555	385,098,741	382,151,246	314,269,145	371,390,747	1,109,082,737	659,401,504
Fraction Reads in Cells	57.40%	81.80%	81.60%	87.60%	85.30%	88.90%	87.40%	83.30%
Mean Reads per Cell	83,114	60,863	61,527	67,506	45,421	56,554	66,672	64,978
Median UMI Counts per Cell	4,836	7,564	5,873	9,096	7,113	10,262	6,012	4,820
Total Genes Detected	19,861	21,665	20,826	20,151	21,110	20,772	27,937	26,306
Median Genes per Cell	1,584	2,516	2,188	2,907	2,444	3,189	2,113	1,598

B

Before quality control **After quality control**

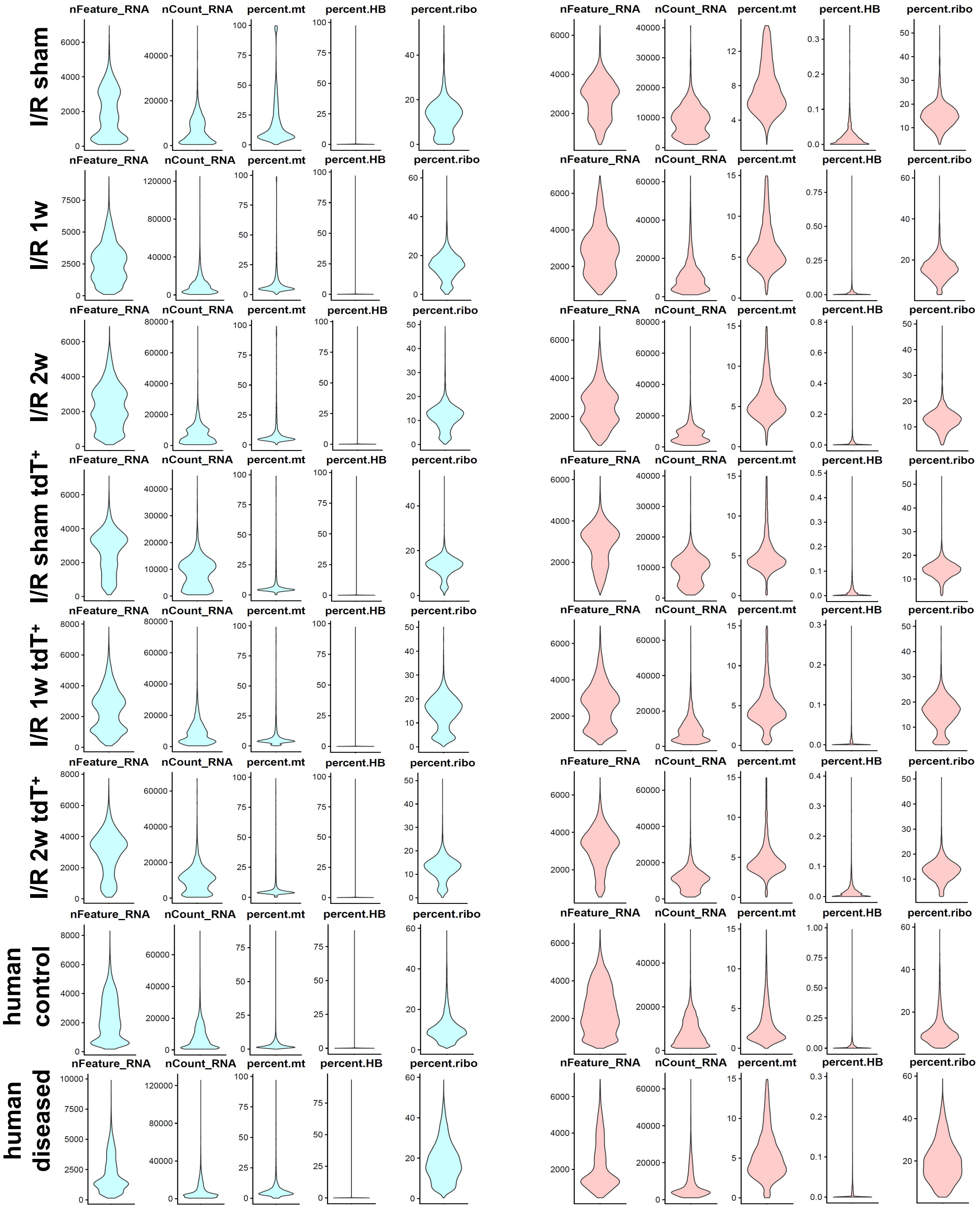

Figure S2. Quality control of single-cell RNA sequencing. (A) Basic quality control metrics of indicated datasets. **(B)** Distribution violin plot for the number of total genes (nFeature_RNA), UMIs (nCount_RNA), percent of mitochondrial genes (percent.mt), hemoglobin genes (percent.HB) and ribosome genes (percent.ribo) detected from each dataset before and after filtering. Cells expressing 400~7000 genes, >1000 UMIs, <1% hemoglobin genes, >3% ribosome genes and <15% mitochondrial genes were filtered for further analyses.

Fig. S3

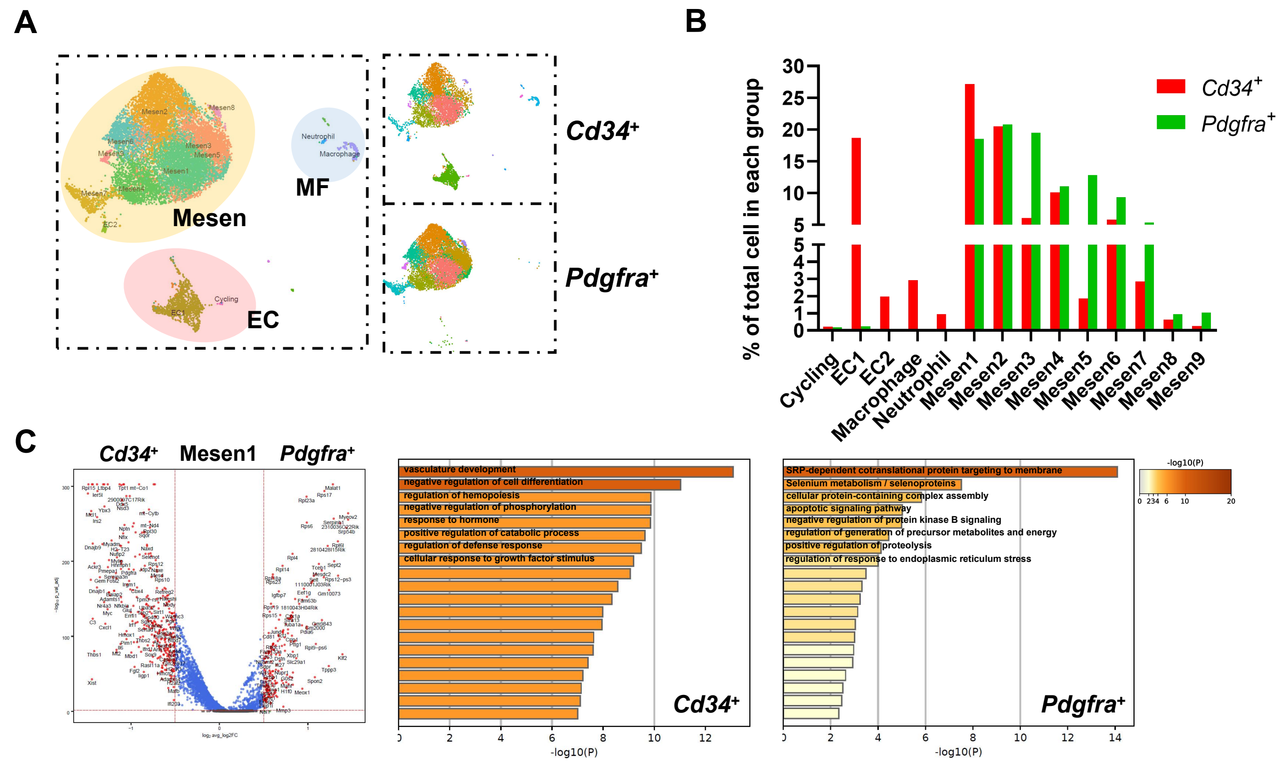

Figure S3. *Cd34*-lineage cells exhibited preserved property of promoting angiogenesis in I/R injury. (A) UMAP plot displaying 14 color-coded cell clusters (3 major cell types) in integrated data by merging sham *Cd34*-lineage cell dataset (*Cd34*⁺) and a public dataset of GFP⁺ cells from the sham group of *Pdfgra*^{eGFP} mice (*Pdgfra*⁺) [7], with corresponding cell clusters at different datasets, n=14,064 cells. **(B)** Bar chart showing the proportion of each cell clusters between *Cd34*⁺ and *Pdgfra*⁺ cells. **(C)** Volcano plots showing differentially expressed genes (DEGs) of cells from Mesen1 between *Cd34*⁺ and *Pdgfra*⁺ cells, with corresponding pathway enrichment analyses using significant DEGs showing enriched biological processes. MF, macrophage. EC, endothelial cell. Mesen, mesenchymal cell. tdT, tdTomato.

Fig. S4

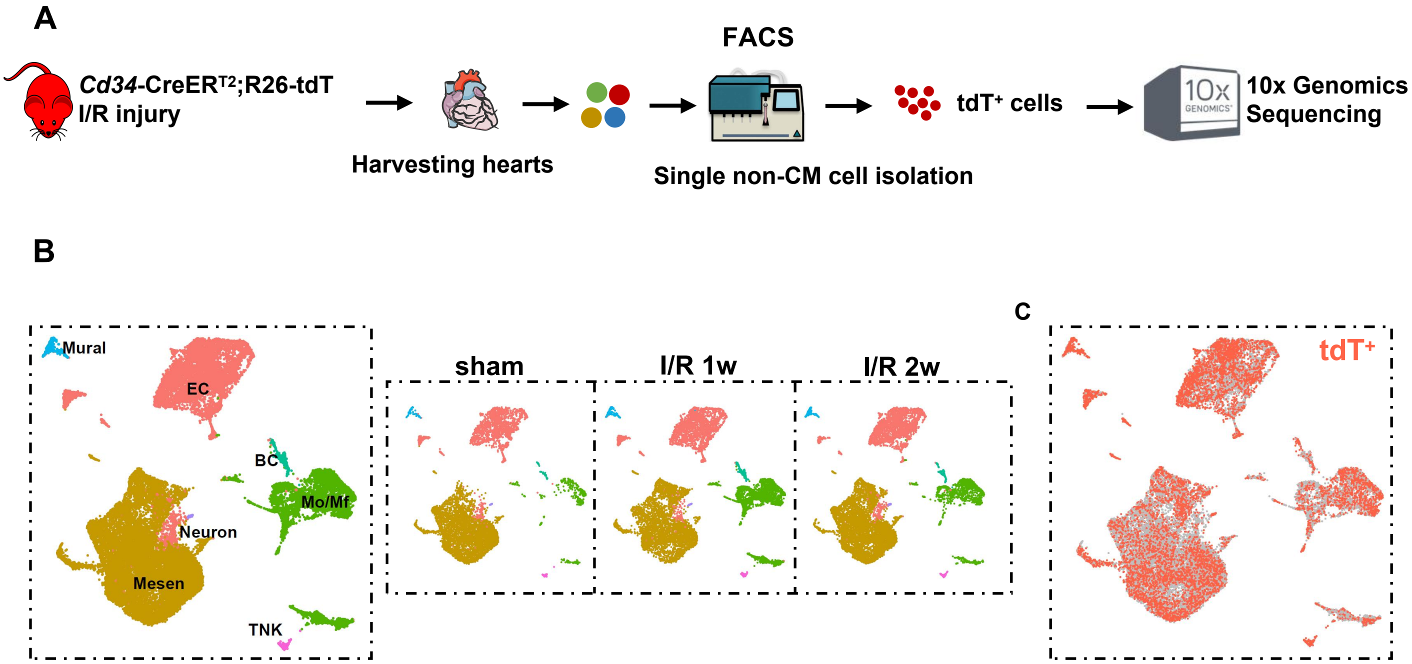

Figure S4. Study of Cd34-lineage cells in I/R injury using lineage tracing models at single-cell level. (A) Schematic depicting heart tissue harvested from *Cd34*-CreER^{T2};R26-tdT (Cre/TDT) mice for scRNA-seq. I/R injury and sham operations were performed and heart tissue was harvested at indicated time points. Single cells were then isolated from the left ventricle, sorted as tdT^{+} cells according to the expression of lineage marker tdT by fluorescence-activated cell sorting (FACS) and subjected for scRNA-seq (n=3 mice per group). **(B)** The integrated scRNA-seq dataset visualized by UMAP plot displaying the 7 color-coded major cell types from total-cell and tdT-labeled-*Cd34*-lineage-cell datasets from sham, 1- and 2-week post-I/R heart tissue, n=26,320 cells. Cell clustering at different time points was shown on the right. **(C)** Feature plot showing the distribution of tdT^{+} cells across all cell clusters. BC, B cell. TNK, T and natural killer (NK) cells. Mo/Mf, monocyte/macrophage. EC, endothelial cell. Mesen, mesenchymal cell. tdT, tdTomato.

Fig. S5

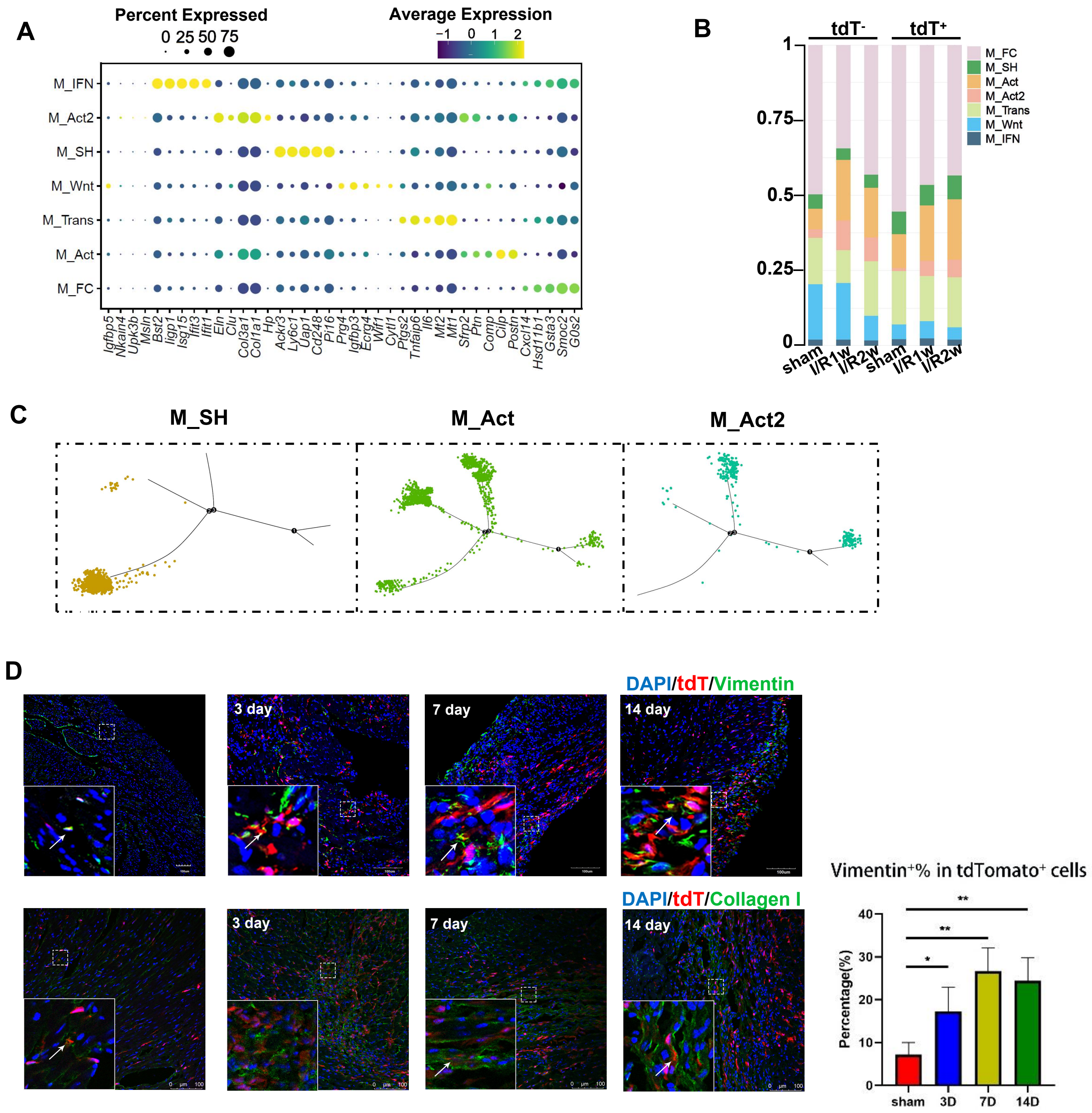

Figure S5. Contribution of *Cd34*-lineage mesenchymal cells in I/R injury. (A) Dot plot displaying average scaled expression levels of top 5 DEGs across identified mesenchymal cell sub-clusters. **(B)** Bar charts showing the proportion of mesenchymal sub-clusters among different time points. **(C)** Monocle trajectory analyses over pseudotime showing cell distribution of M_SH, M_Act and M_Act2 sub-clusters separately. **(D)** Representative cross-sections of Cre/TDT ventricles stained with tdT and Vimentin/ Collagen I in border zones at the indicated time point, with magnification of the boxed region, arrows indicated co-staining cells. Quantification of the percentages of Vimentin+ cells in tdTomato+ cells at the indicated time points by immunofluorescence was shown on the right panel. N=3 mice in the sham group and N=4 mice in the I/R 3d, 7d, 14d groups, *$p<0.05$, **$p<0.01$, by one-way ANNOVA test. Mesen, mesenchymal cell. IFN, interferon stimulated. Act, activated. SH, Sca-1 high. tdT, tdTomato.

Fig. S6

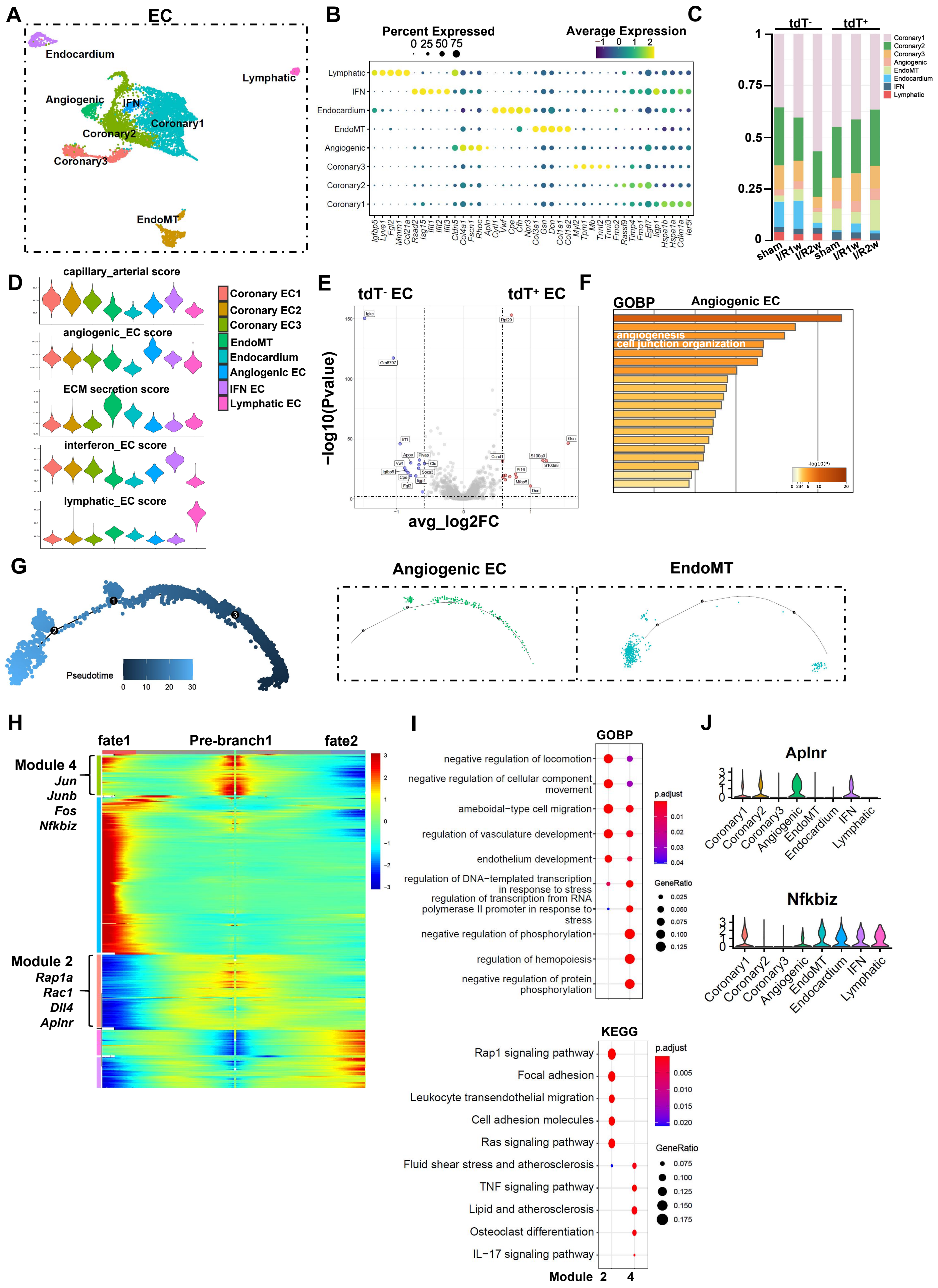
A
EC
Endocardium
Lymphatic
Angiogenic
IFN
Coronary1
Coronary2
Coronary3
EndoMT
B
Percent Expressed
0 25 50 75
Average Expression
−1 0 1 2
Lymphatic
IFN
Endocardium
EndoMT
Angiogenic
Coronary3
Coronary2
Coronary1
C
tdT-
tdT+
Coronary1
Coronary2
Coronary3
Angiogenic
EndoMT
Endocardium
IFN
Lymphatic
sham
I/R1w
I/R2w
D
capillary_arterial score
angiogenic_EC score
ECM secretion score
interferon_EC score
lymphatic_EC score
Coronary EC1
Coronary EC2
Coronary EC3
EndoMT
Endocardium
Angiogenic EC
IFN EC
Lymphatic EC
E
tdT- EC
tdT+ EC
-log10(Pvalue)
avg_log2FC
F
GOBP
Angiogenic EC
angiogenesis
cell junction organization
-log10(P)
G
Pseudotime
Angiogenic EC
EndoMT
H
fate1
Pre-branch1
fate2
Module 4
Jun
Junb
Fos
Nfkbiz
Module 2
Rap1a
Rac1
Dll4
Aplnr
I
GOBP
negative regulation of locomotion
negative regulation of cellular component movement
ameboidal−type cell migration
regulation of vasculature development
endothelium development
regulation of DNA−templated transcription in response to stress
regulation of transcription from RNA polymerase II promoter in response to stress
negative regulation of phosphorylation
regulation of hemopoiesis
negative regulation of protein phosphorylation
p.adjust
GeneRatio
KEGG
Rap1 signaling pathway
Focal adhesion
Leukocyte transendothelial migration
Cell adhesion molecules
Ras signaling pathway
Fluid shear stress and atherosclerosis
TNF signaling pathway
Lipid and atherosclerosis
Osteoclast differentiation
IL−17 signaling pathway
Module 2 4
J
Aplnr
Nfkbiz
Coronary1
Coronary2
Coronary3
Angiogenic
EndoMT
Endocardium
IFN
Lymphatic

Figure S6. Contribution of *Cd34*-lineage endothelial cells in I/R injury. **(A)** UMAP plot showing 8 color-coded sub-clusters from the endothelial cell (EC) population in I/R injury, n=6,679 cells. **(B)** Dot plot displaying average scaled expression levels of top 5 DEGs across identified EC sub-clusters. **(C)** Bar charts showing the proportion of EC sub-clusters among different time points. **(D)** Violin plots showing expression scores (Z-scores) of capillary-arterial, angiogenic, ECM secretion, interferon stimulated and lymphatic genes across EC sub-clusters. **(E)** Volcano plot showing DEGs between tdT^+ ECs and tdT^- ECs. **(F)** Go enrichment biological process (GOBP) analysis of the angiogenic EC cluster using marker genes to show putative functions. **(G)** Monocle trajectory analyses of ECs showing cell distribution over pseudotime. Cell distribution of angiogenic EC and EndoMT sub-clusters was separately displayed on the right. **(H)** Heatmap of the significantly changed genes via BEAM function from monocle in the branch point 1 in Fig. 1o, with representative up-regulated genes listed from 2 pre-branch gene modules (Module 2 and 4). **(I)** GOBP and Kyoto Encyclopedia of Genes and Genomes (KEGG) analyses of gene Module 2 and 4 indicating distinct biological functions and pathways. **(J)** Violin plots showing representative genes of Module 2 (*Aplnr*) and 4 (*Nfkbiz*) among EC sub-clusters. EndoMT, endothelial-to-mesenchymal transition. IFN, interferon stimulated. EC, endothelial cell. tdT, tdTomato.

Fig. S7

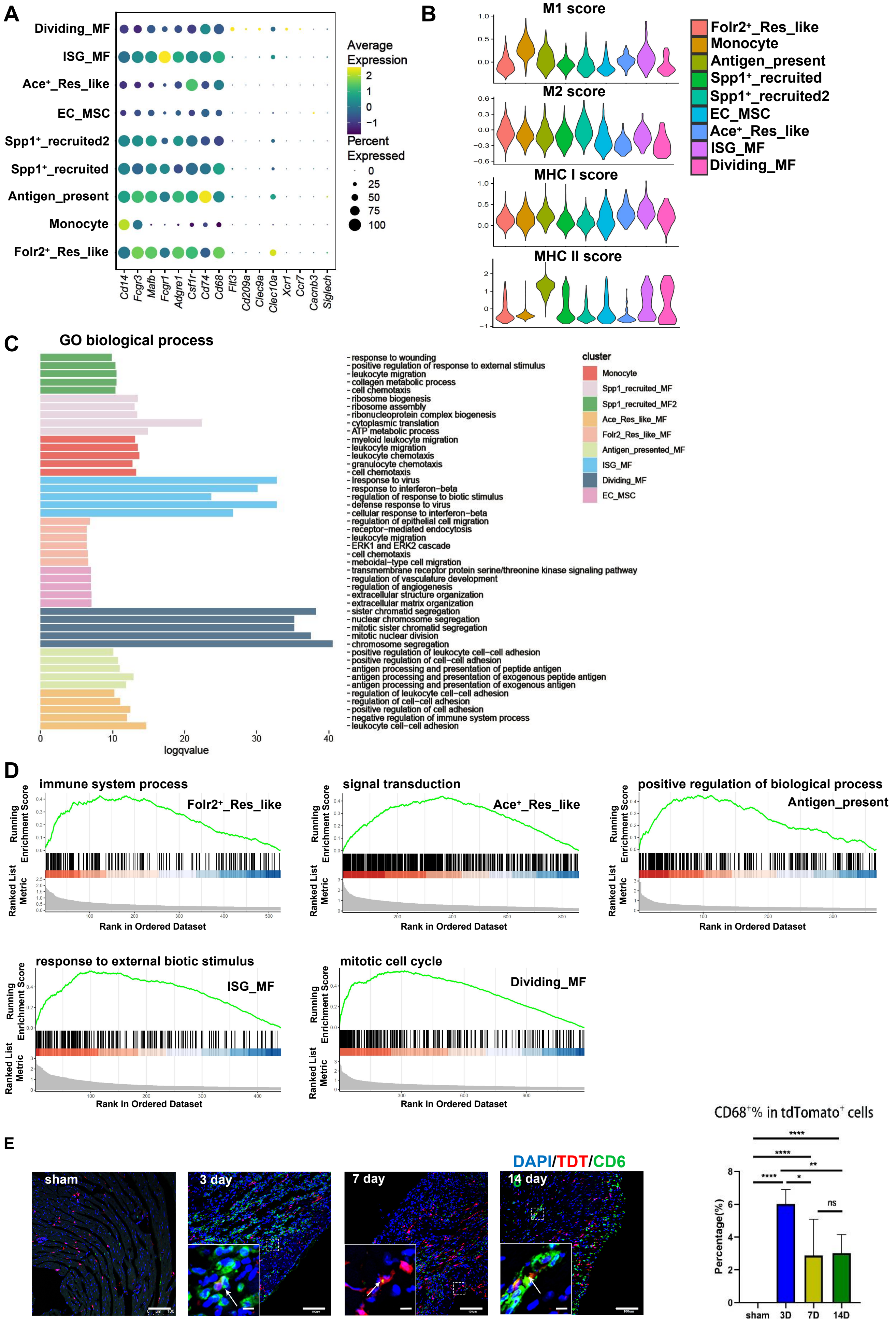
A
Dividing_MF
ISG_MF
Ace⁺_Res_like
EC_MSC
Spp1⁺_recruited2
Spp1⁺_recruited
Antigen_present
Monocyte
Folr2⁺_Res_like
Cd14
Fcgr3
Mafb
Fcgr1
Adgre1
Csf1r
Cd74
Cd68
Flt3
Cd209a
Clec9a
Clec10a
Xcr1
Ccr7
Cacnb3
Siglech
Average Expression
2
1
0
−1
Percent Expressed
0
25
50
75
100
B
M1 score
M2 score
MHC I score
MHC II score
Folr2⁺_Res_like
Monocyte
Antigen_present
Spp1⁺_recruited
Spp1⁺_recruited2
EC_MSC
Ace⁺_Res_like
ISG_MF
Dividing_MF
C
GO biological process
response to wounding
positive regulation of response to external stimulus
leukocyte migration
collagen metabolic process
cell chemotaxis
ribosome biogenesis
ribosome assembly
ribonucleoprotein complex biogenesis
cytoplasmic translation
ATP metabolic process
myeloid leukocyte migration
leukocyte migration
leukocyte chemotaxis
granulocyte chemotaxis
cell chemotaxis
response to virus
response to interferon−beta
regulation of response to biotic stimulus
defense response to virus
cellular response to interferon−beta
regulation of epithelial cell migration
receptor−mediated endocytosis
leukocyte migration
ERK1 and ERK2 cascade
cell chemotaxis
meboidal−type cell migration
transmembrane receptor protein serine/threonine kinase signaling pathway
regulation of vasculature development
regulation of angiogenesis
extracellular structure organization
extracellular matrix organization
sister chromatid segregation
nuclear chromosome segregation
mitotic sister chromatid segregation
mitotic nuclear division
chromosome segregation
positive regulation of leukocyte cell−cell adhesion
positive regulation of cell−cell adhesion
antigen processing and presentation of peptide antigen
antigen processing and presentation of exogenous peptide antigen
antigen processing and presentation of exogenous antigen
regulation of leukocyte cell−cell adhesion
regulation of cell−cell adhesion
positive regulation of cell adhesion
negative regulation of immune system process
leukocyte cell−cell adhesion
logqvalue
cluster
Monocyte
Spp1_recruited_MF
Spp1_recruited_MF2
Ace_Res_like_MF
Folr2_Res_like_MF
Antigen_presented_MF
ISG_MF
Dividing_MF
EC_MSC
D
immune system process
Folr2⁺_Res_like
signal transduction
Ace⁺_Res_like
positive regulation of biological process
Antigen_present
response to external biotic stimulus
ISG_MF
mitotic cell cycle
Dividing_MF
Running Enrichment Score
Ranked List Metric
Rank in Ordered Dataset
E
sham
3 day
7 day
14 day
DAPI/TDT/CD6
CD68⁺% in tdTomato⁺ cells
Percentage(%)
****
****
**
****
*
ns
sham
3D
7D
14D

Figure S7. Contribution of *Cd34*-lineage endothelial cells in I/R injury. (A) Dot plot displaying average scaled expression of selected marker genes to define cell sub-clusters including monocyte (*Cd14)*, macrophage (*Mafb*, *Fcgr1*, *Adgre1*) and dendritic cells (DCs; *Flt3*, *Cd209a*, *Clec9a*, *Clec10a*, *Xcr1*, *Ccr7*, *Cacnb3*, *Siglech*). Dot size reflects the percentage of cells expressing the selected gene in each cell cluster. **(B)** violin plots showing expression scores of M1, M2, MHCI and MHC II genes across all sub-clusters. **(C)** GOBP analyses showing top 5 enriched pathways of each sub-cluster according to their marker genes. **(D)** GSEA analyses of enriched pathways in each sub-cluster other than recruited macrophages. **(E)** Representative cross-sections of Cre/TDT ventricles stained with tdTomato and CD68 in border zone at the indicated time point, with magnification of the boxed region. Quantification of the percentages of $CD68^+$ cells in $tdTomato^+$ at the indicated time points by immunofluorescence was shown on the right. Scar bar: 100μm and 20 μm in magnification. N=3 mice in sham group and N=4 mice in the I/R groups; *P<0.05;**P<0.01; ****P<0.0001, by one-way ANNOVA test.

Fig. S8

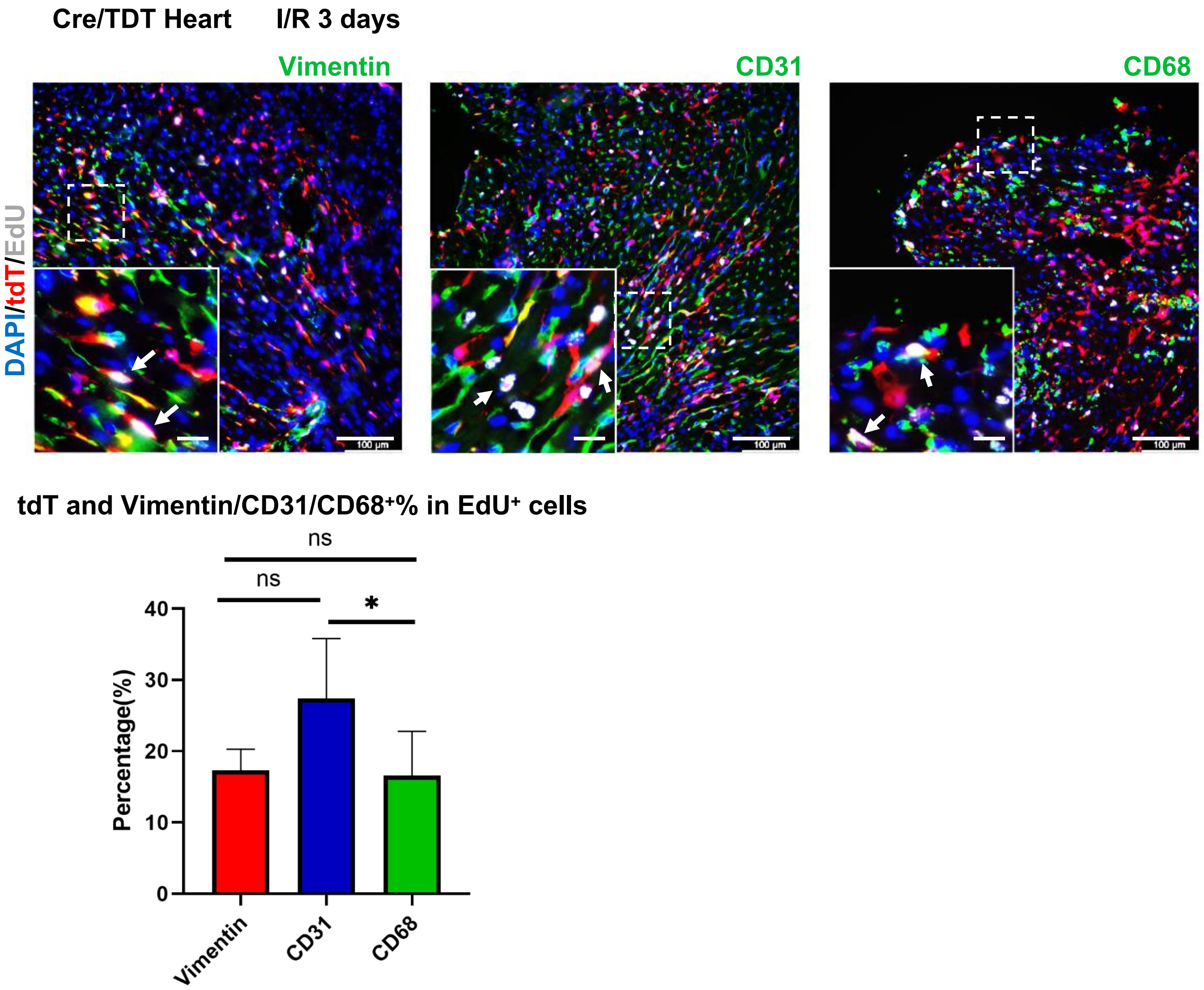

Figure S8. Proliferating property of *Cd34*-lineage cells in I/R injury. Representative cross-sections of Cre/TDT ventricles stained with tdT, Vimentin/CD31/CD68 and EdU. Magnification in each image was the magnification of the boxed region, arrows indicated co-staining cells. Quantification of the percentage of proliferating cells was shown below. Scale bars, 100 μm, and 20 μm in magnification images. tdT, tdTomato.

Fig. S9

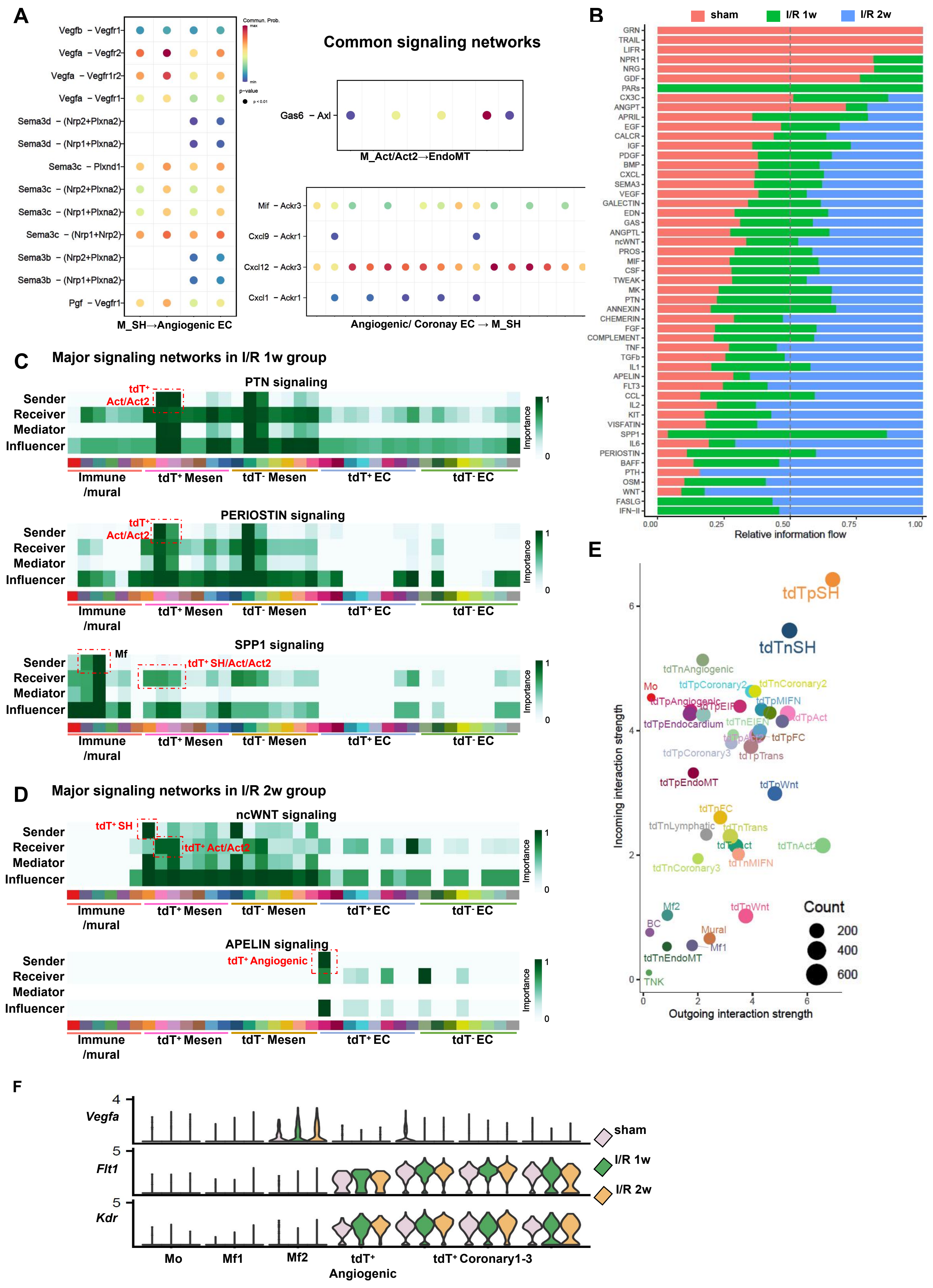
A
M_SH→Angiogenic EC
Common signaling networks
Gas6 - Axl
M_Act/Act2→EndoMT
Angiogenic/ Coronay EC → M_SH
B
sham
I/R 1w
I/R 2w
Relative information flow
C
Major signaling networks in I/R 1w group
PTN signaling
PERIOSTIN signaling
SPP1 signaling
Sender
Receiver
Mediator
Influencer
Importance
Immune /mural
tdT+ Mesen
tdT- Mesen
tdT+ EC
tdT- EC
D
Major signaling networks in I/R 2w group
ncWNT signaling
APELIN signaling
tdT+ Angiogenic
E
Incoming interaction strength
Outgoing interaction strength
Count
F
Vegfa
Flt1
Kdr
Mo
Mf1
Mf2
tdT+ Angiogenic
tdT+ Coronary1-3

Figure S9. Intercellular communicating patterns in I/R injury. (A) Summary dot plots showing common signaling networks between certain mesenchymal and EC sub-clusters via certain ligand-receptor pairs in the whole process of I/R injury. **(B)** Selected signaling pathway networks that were strongly active in either time point based on the diferences of overall information flow as predicted by CellChat. The overall information flow of a signaling network was calculated by summarizing all the communication probabilities in that network. **(C)** Heatmaps of major signaling networks in the I/R 1w group showing relative importance of each cell group based on the computed four network centrality measures of each signaling network. **(D)** Heatmaps of major signaling networks in the I/R 2w group showing relative importance of each cell group. **(E)** Dot plots displaying the overall incoming/ outgoing interaction strength in intercellular communication analyses, indicating the cell-cell communicating activities of each cell cluster. **(F)** Violin plots displaying expression profile of major VEGF signaling ligand-receptor gene pairs (*Vegfa-Flt1*, *Vegfa-Kdr*) between Mo/Mf clusters and tdT^{+} angiogenic/ coronary ECs among different time points after I/R injury. Mo, monocyte. Mf, macrophage. Mesen, mesenchymal cell. EndoMT, endothelial-to-mesenchymal transition. IFN, interferon stimulated. EC, endothelial cell. tdT, tdTomato. tdTp, tdT positive. tdTn, tdT negative.

Fig. S10

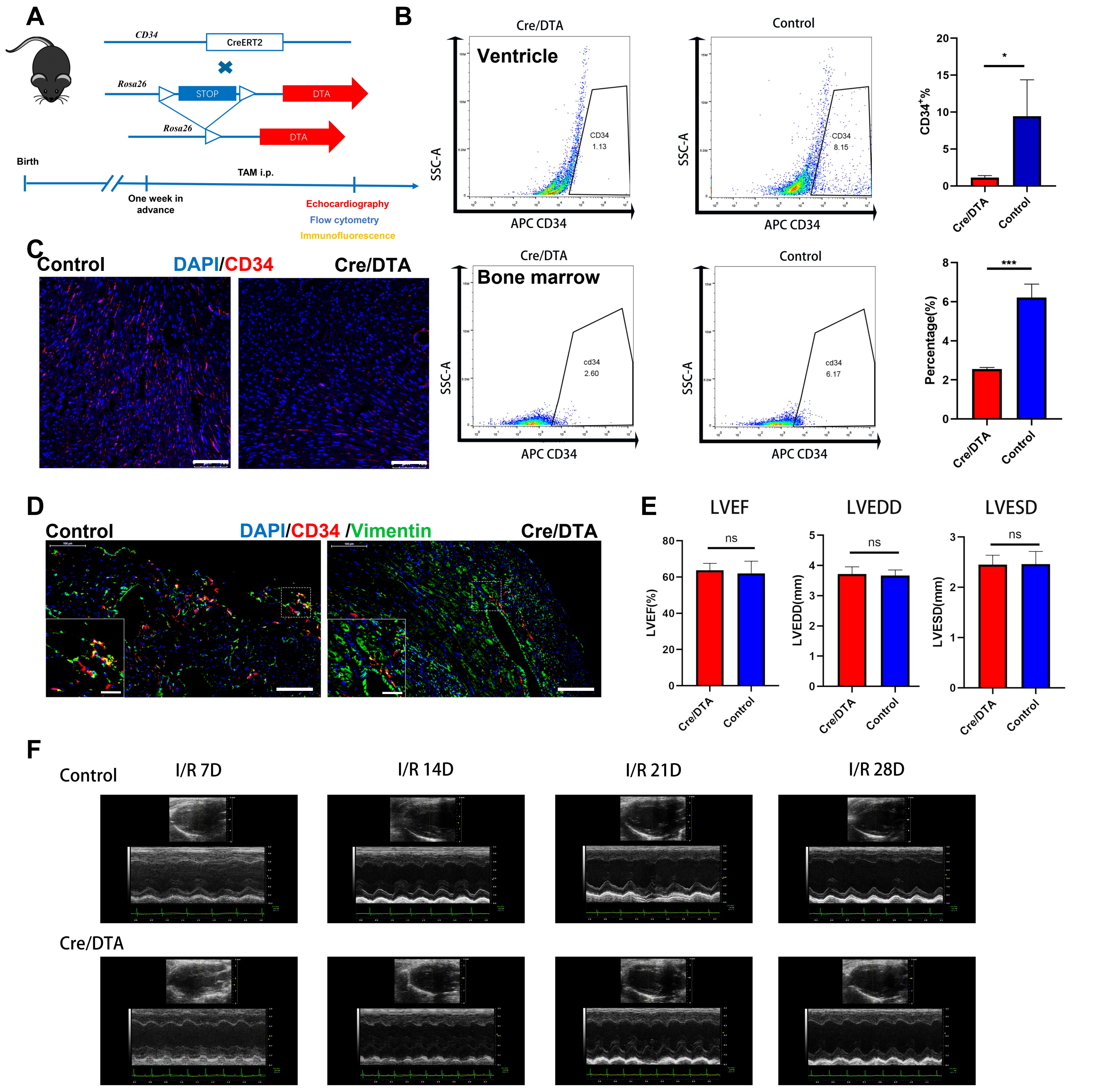

Figure S10. The effect of CD34⁺ cell ablation on I/R injury. (A) Schematic of the *Cd34*-CreER^{T2};R26-DTA (Cre/DTA) mice. Under the administration of tamoxifen the LoxP flanked STOP cassette was recombinated off to induce a diphtheria toxin (DT) induced CD34^{+} cell ablation. Experimental scheme whereby Cre/DTA mice were given tamoxifen 1 week before heart harvesting. **(B)** Representative flow cytometry analyses of freshly isolated ventricles cells (upper) and bone marrow cells (below) from Cre/DTA mice or control mice staining with APC-conjugated CD34 antibody, with quantification of CD34^{+} cells among all isolated cells between two genotypes. N=3 mice per group. **(C)** Representative cross-sections of Cre/DTA or control ventricles staining CD34. Cre/DTA mice with pure oil administration were used as control. **(D)** Representative cross-sections of Cre/DTA and control ventricles staining CD34 with vimentin in border zones at 4-week after I/R injury, with magnification of the boxed region. **(E)** Echocardiographic measurements of LVEF, LVEDD and LVESD in Cre/DTA or control mice under normal condition. N=7 mice per group. **(F)** Representative echocardiogram of Cre/DTA and control mice after I/R injury at different time point. Data was shown as mean ± SEM, ns, not significant, *p<0.05, ***p<0.001, by student's t test. Scale bars, 100μm and 20 μm in magnification.

Fig. S11

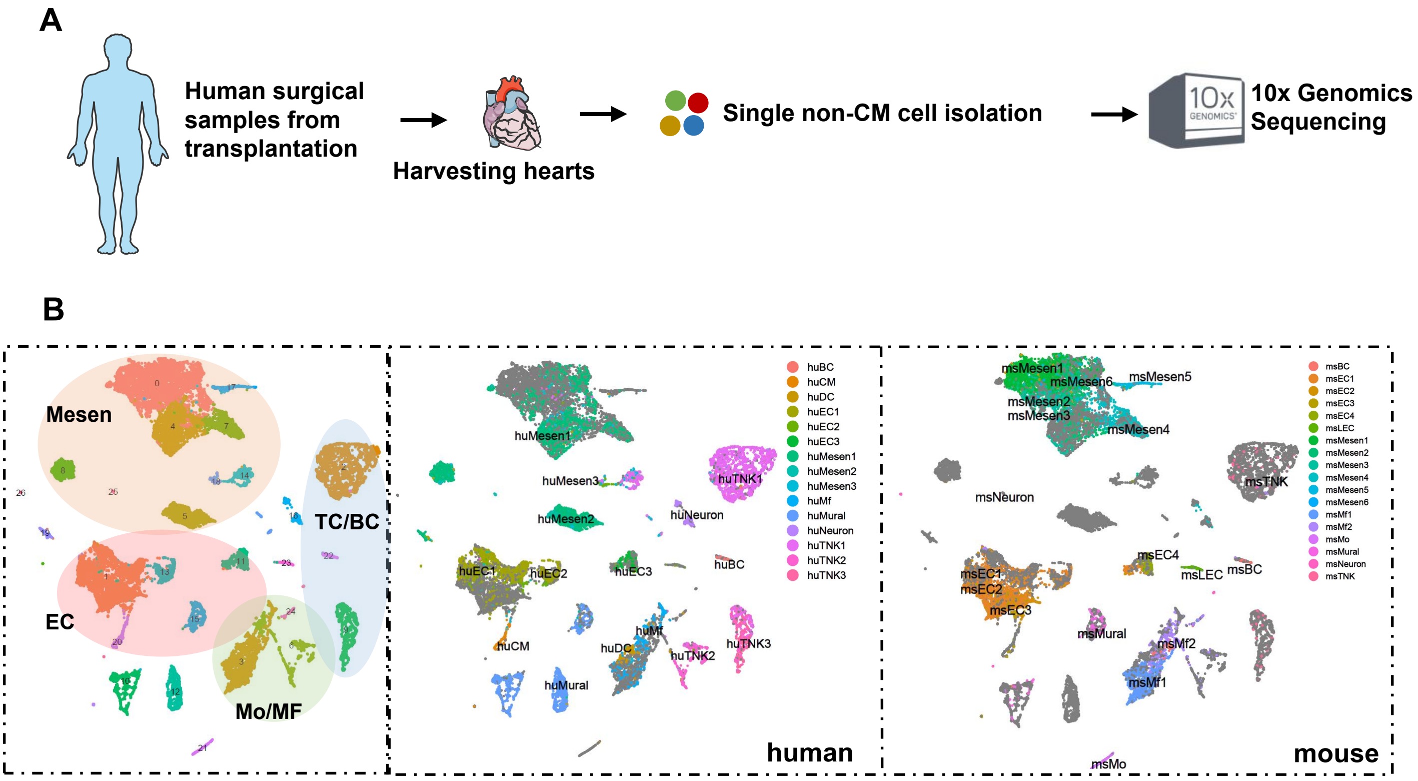

Figure S11. Validation of CD34⁺ cells in human heart. (A) Schematic depicting heart tissue harvested from human. Left ventricle tissue was obtained from patients undergoing heart transplantation and enzymatically digested for scRNA-seq. **(B)** UMAP plots exhibiting integrated and separate human and mouse single-cell cardiac non-CM clustering analysed by LIGER, color-coded by cell types. BC, B cell. TC, T cells. Mo, monocyte. MF, macrophage. EC, endothelial cell. Mesen, mesenchymal cell.
