# Supplementary material for: Multilineage contribution of CD34+ cells in cardiac remodeling after ischemia/reperfusion injury
Source: Basic Res Cardiol. 2023 May 5;118(1):17. doi: 10.1007/s00395-023-00981-8 (PMC10163140; doi:10.1007/s00395-023-00981-8)
Supplement: Supplementary file 8 — Supplementary file8 (DOCX 67 KB) [file 395_2023_981_MOESM8_ESM.docx]

**Supplemental methods**

**Multilineage Contribution of CD34^+^ Cells in Cardiac Remodeling after Ischemia/Reperfusion Injury**

**Running title**: CD34^+^ Cells in Myocardial I/R Injury

Jun Xie ^1†^, Liujun Jiang ^2†^, Junzhuo Wang ^1†^, Yong Yin ^1^, Ruilin Wang ^2^, Ting Chen ^2,3^, Zhichao Ni ^2^, Shuaihua Qiao ^1^, Hui Gong ^2^, Biao Xu ^1^*, Qingbo Xu ^2^*

^1^Department of Cardiology, Drum Tower Hospital, State Key Laboratory of Pharmaceutical Biotechnology, Medical School of Nanjing University, Nanjing, China;

^2^Department of Cardiology, the First Affiliated Hospital, Zhejiang University School of Medicine, Hangzhou, China;

^3^Alibaba-Zhejiang University Joint Research Center of Future Digital Healthcare, Hangzhou, China.

*Corresponding author. Biao Xu, MD, PhD, Department of Cardiology, Drum Tower Hospital, State Key Laboratory of Pharmaceutical Biotechnology, Medical School of Nanjing University, No. 321 Zhongshan Road, Nanjing, 210008, Jiangsu, China. Phone and Fax: +86 25-68182812, E-mail: xubiao62@nju.edu.cn. And Qingbo Xu, MD, PhD, Department of Cardiology, the First Affiliated Hospital, Zhejiang University School of Medicine, 79 Qingchun Road, Hangzhou, Zhejiang Province, P.R.China, 310003. Tel: +86 571-87236500, Fax: +86 571 4008306430, Email: qingbo_xu@zju.edu.cn.

^†^ These authors contributed equally to this work.

**Animal procedures**

This study used 8- to 10-week-old male mice and randomly allocated them to different experimental groups. The number of mice used for each experiment is indicated in the figure legends. The animals were fed a standard laboratory diet with free access to food and water and kept in a temperature- (22 °C ± 1°C) and humidity-controlled (65%–70%) room, with a 12-h light–dark cycle. All mice generated or purchased were housed in the Nanjing Drum Tower Hospital (Jiangsu, China) for at least 1 week before use.

The mice were injected with tamoxifen (T5648, Sigma) dissolved in corn oil intraperitoneally at a dose of 20 mg/kg body weight per day for 1 week to induce the activity of the CreER^T2^ protein and further perform the genetic labeling of CD34^+^ cells. Tamoxifen was administrated to *Cd34*-CreER^T2^; R26-DTA/tdTomato mice 1 week before the surgery and once a week after the surgery to achieve diphtheria toxin (DT)-induced CD34^+^ cell apoptosis.

MI or myocardial I/R model was performed. Briefly, the mice were first anesthetized with global anesthesia by placing them in an anesthesia induction box connected to a vaporizer set to 3.0 vol% of isoflurane with an 100% oxygen flow of 1 L/min. During the surgical procedure, anesthesia was maintained at 1.5%–2% isoflurane in 0.5–1 L/min 100% oxygen, and ventilated with 3-cm H_2_O positive-end expiratory pressure using a small-animal ventilator. The adequacy of anesthesia was monitored using corneal and withdrawal reflexes. The ventilation frequency was kept at 110 breaths/min with a tidal volume between 135 and 150 μL. After ventilation, a thoracotomy at the fourth intercostal space was performed to expose the heart clearly. For MI surgery, left anterior descending (LAD) coronary artery was permanently ligated with silk suture. For I/R surgery, the LAD coronary artery was temporarily ligated and the silk suture was released for reperfusion after 45 min. In the sham-operated group, the LAD coronary artery was not ligated.

The mice were euthanized using carbon dioxide (CO_2_) at indicated time points after ischemic models following the NIH guidelines, with minimal stress to the animals. Briefly, a cage containing three to five mice was placed in a separate 20-L volume chamber. Compressed 99.99% CO_2_ gas in a cylinder was connected to and introduced into the chamber with a flow rate of 10 L/min. The mice were all unconscious in 3 min, with no spontaneous breathing. After another 1 min of CO_2_ flow, the mice were checked again to confirm the absence of respiration, and their eye color faded with no pupillary response to light. The mice were then removed from the cage, the chests were cut open, and the hearts were perfused through right ventricular puncture, with certain solutions according to different further experiments. The hearts were then removed and harvested for single-cell sequencing and flow cytometric analyses [Dulbecco's modified Eagle medium (DMEM)] containing 3% fetal bovine serum (FBS) for perfusion) or immunostaining [phosphate-buffered saline (PBS) for perfusion], and the mice were confirmed dead because of the removal of hearts.

**EdU in a proliferation assay**

The mice were injected with 5-ethynyl-2′-deoxyuridine (EdU) intraperitoneally at a dose of 20 mg/kg body weight per day. After 24 h of EdU injection, the heart samples were collected. EdU detection was carried out before immunocytochemical staining using Cell-LightTM Apollo 643 Stain Kit (C10371-2, RiboBio).

**Histological studies**

The hearts were harvested, embedded in paraffin, and cut into 5-μm-thick sections. The sections were stained with Masson trichrome following the manufacturer’s protocols. The images were obtained from the base, mid-ventricle, and apical regions of the hearts. We selected different cross-sections and calculated the ratio of the blue-stained (collagen) area to the total ventricular area using the Image-Pro Plus software to assess interstitial fibrosis. The scar tissue area was also calculated as a fraction of left ventricular surface area occupied by the scar tissue using Image-Pro Plus software [[11](#_ENREF_11)]. The severity of ﬁbrosis was classiﬁed as “moderate” if the sample showed 20%–40% ﬁbrotic area, and “mild” if the sample showed <20% ﬁbrotic area.

**Immunofluorescence staining**

Human and mouse samples were fixed in 4% paraformaldehyde for 2–3 h, and washed with PBS for 20 min. Then, the samples were dehydrated in PBS containing 30% sucrose overnight at 4°C before embedding the samples in Tissue-Tek OCT for cryosectioning. The frozen sections (8–10 μm) were incubated in blocking buffer containing 10% goat serum and then incubated with primary antibody diluted in blocking buffer overnight at 4°C. The sections were incubated with a secondary antibody and 4′,6-diamidino-2-phenylindole incubated in PBS for 2 h at room temperature after rinsing three times with PBS. The images were captured. A semi-quantitative analysis of immunofluorescence was performed using the Image-Pro Plus software. The antibodies used in this study were as follows: anti-vimentin antibody (Abcam, ab45939, 1:200), anti-CD31 antibody (BD Pharmingen, 550274, 1:200), anti-CD45 antibody (eBioscience, 14-5981-81, 1:100), anti-Ly6a (Sca-1) antibody (eBioscience, 14-0451-81, 1:100), anti-IFITM3 antibody (Proteintech, 11714-1-AP, 1:200), anti-IFITM2 antibody (Proteintech, 12769-1-AP, 1:20), anti-IFITM1 antibody (Proteintech, 60074-1-lg, 1:50), anti-periostin antibody (Proteintech, 19899-1-AP, 1:100), anti-CD34 antibody (Novus, DDX0362P-100, 1:150), anti-Ki67 antibody (Abcam, ab16667, 1:500), goat anti-rat immunoglobulin G (IgG) (H+L) Alexa Fluor 488 (Jackson ImmunoResearch, 112-545-167, 1:200), goat anti-rabbit IgG (H+L) Alexa Fluor 488 (Jackson ImmunoResearch, 112-545-144, 1:200), goat anti-rat IgG (H+L) Cy3 (Jackson ImmunoResearch, 112-165-167, 1:200), and donkey anti-rabbit IgG (H+L) Cy5 (Jackson ImmunoResearch, 715-175-151, 1:200).

**Single-cell dissociation from heart tissue**

The samples from human and mouse hearts were processed as follows. Briefly, the left ventricles were first washed with PBS, minced into small pieces (approximately 1 mm^3^) on ice, and enzymatically digested in the DMEM + 0.5 mg/mL Liberase medium (Roche) for 20–30 min at 37°C. After digestion, the samples were sieved through a 40-µm cell strainer and centrifuged at 300*g* for 5 min. After the supernatant was removed, noncardiomyocyte sample pelleted cells were suspended in red blood cell lysis buffer (Miltenyi Biotec) to lyse the red blood cells. The single-cell suspension was further enriched using a MACS Dead Cell Removal Kit (Miltenyi Biotec). The enriched single cells were then stained with 7-AAD Viability Staining Solution (FcmacsBiotech) for viability assessment. Subsequently, the cell pellet was suspended in relevant solutions for further experiments.

**Flow cytometry**

For dissociated heart cells, the dissolved cell pellet was suspended in PBS with 5% FBS. For bone marrow and blood cells, the harvested cells were incubated with red blood cell lysis buffer to lyse red blood cells and passed through a 40-μm cell strainer to obtain single-cell suspensions. For the staining of certain cells, the cells were stained with conjugated antibodies (1 µL per 10^6^ cells) for 30 min at 4℃. The antibody used in this study was allophycocyanin anti-mouse CD34 (BioLegend, 128612). The corresponding control isotype antibodies were used as control. The cells were then washed with PBS and resuspended in PBS containing 5% FBS for flow cytometric analysis. The FlowJo v10 software (Tree Star) was used to analyze the flow cytometric data.

**Single-cell RNA sequencing**

After complete digestion, the cell pellet from mouse or human samples was suspended in PBS and stained with 7-AAD for 20 mins on ice. After PBS washing, the cells were resuspended in PBS and single live cells (7-AAD^-^) were sorted into PBS with 0.04% bovine serum albumin using a BD FACS ARIA II flow cytometer (BD Biosciences). For *Cd34*-lineage tdTomato^+^ cells, tdTomato autofluorescence was detected using the flow cytometer and single live tdTomato^+^ cells were sorted for further sequencing. A Chromium Single Cell 3' Reagent Kit v2 (mouse) or v3 (human) chemistry (10× Genomics) was used, and a standard protocol was followed. The final libraries were quantified using the Qubit high-sensitivity DNA assay (Thermo Fisher Scientific), and the size distribution of the libraries was determined using a high-sensitivity DNA chip on a Bioanalyzer 2200 (Agilent). All libraries were sequenced using an Illumina sequencer (Illumina, CA, USA) on a 150-bp paired-end run. For the humans, the live cells were sorted using a Dead Cell Removal Kit (Miltenyi Biotec, 130-090-101) and subjected to scRNA-seq on the 10× Genomics platform using a Chromium Single Cell 3' Reagent Kit v3 chemistry. The scRNA-seq of mouse samples was conducted at the NovelBio Bio-Pharm Technology Co., Ltd, while that of human samples at the Shanghai OE Biotech Co., Ltd.

**Single-cell RNA statistical analyses**

Single-cell RNA-sequencing raw data were processed using Cell Ranger (version 6.0); aligned reads and gene-barcode matrices were then generated from FASTQ files, including Read 1, Read 2, and i7 index. Briefly, “Cellranger mkfastq” was used to demultiplex raw data and generate FASTQ files, which were further processed by “Cellranger count” to align reads to the mouse reference to count the number of barcode and UMI, and to generate feature-barcode matrices. Median reads and genes detected per cell are summarized in Fig. S2.

After the aggregation of the samples, further analyses and visualization were performed using the R package Seurat v4.0.5 [[1](#_ENREF_1), [8](#_ENREF_8)]. The analyses were performed with default parameters unless otherwise specified. Briefly, the gene features expressed in at least 10 cells and cells with at least 100 detected genes were kept, followed by filtering cells displaying <400 or >7000 gene features, <1000 UMI counts, >1% hemoglobulin gene counts, and <3% ribosome gene counts, and those having >15% mitochondrial counts (Fig. S2). The data of cells that remained after filtering were normalized and scaled through the “SCTransform” function. The top 3000 highly variable genes were selected. The principle component analysis was then performed on selected highly variable genes, and the first 30 principle components with a suitable resolution were used for cell clustering and uniform manifold approximation and projection visualization. The marker genes upregulated in each cluster were identified by “FindAllMakers” (min.pct = 0.25, logfc.threshold = 0.25). The integrative analysis of different datasets was also performed with Seurat using the “SCTIntegration” method to remove the batch effects. Seurat functions “DimPlot,” “FeaturePlot,” “DotPlot,” “VlnPlot,” and “DoHeatmap” were used for visualization of data. Focused analyses of certain cell clusters were performed using the function “subset.”

We integrated two groups of datasets for the merged analyses of our data with other public datasets. In the first group, we integrated our I/R total-cell datasets with sham, day 7, and day 14 post-MI cardiac interstitial cell datasets from the study by Forte et al. [[4](#_ENREF_4)], to find the difference between I/R and MI. In the second group, we integrated our *Cd34-*lineage datasets with sham *Pdgfra*-GFP^+^ dataset from the study by Farbehi et al. [[3](#_ENREF_3)] to compare the *Cd34*-lineage cells with *Pdgfra*-lineage cells. The integration analyses of the aforementioned datasets were performed with similar parameters as our I/R datasets.

The Wilcoxon test was performed for differential expression analyses among cell types or between different groups, as implemented in the “FindAllMarkers” function of the Seurat package. The Volcano plot was produced using EnhancedVolcano (1.3.5), based on the *P* value and fold change of differentially expressed genes (DEGs). The Venn plot was produced using the software TBtools [[2](#_ENREF_2)].

For gene functional annotation, the Gene Ontology and Kyoto Encyclopedia of Genes and Genomes pathway analyses of DEGs were performed using the R package clusterProfiler [[12](#_ENREF_12)] and the online tool Metascape (metascape.org) [[13](#_ENREF_13)], with DEGs expressed by selected cell clusters.

The pseudotime trajectory analyses were performed with the R package SCORPIUS on the clusters using the suggested workflows and Pearson’s correlation, and Monocle (version 2.16) [[6](#_ENREF_6), [7](#_ENREF_7), [9](#_ENREF_9)] with default settings unless otherwise specified. The genes used for pseudotime ordering were taken from the first 250 (by avg_logFC and *P* value) DEGs identified by function differentialGeneTest, with fullModelFormulaStri set as pseudotime. The DDRTree method was used for dimension reduction and cell ordering along the pseudotime trajectory. The branch analyses were performed using the BEAM function, when presenting the significantly changed (*P* < 0.01) genes in the branch point.

We performed analyses of ligand–receptor pairs among cell clusters using the package CellChat for intercellular communication analyses [[5](#_ENREF_5)]. For each ligand–receptor pair, the CellChat assigned a communication probability value by the law of mass action based on the average expression values of a ligand by one cell group and that of a receptor by another cell group. The statistical significance of communication probability values was assessed using a permutation test, and a *P* value <0.05 indicated a statistically significant difference.

The R package linked inference of genomic experimental relationships (LIGER) [[10](#_ENREF_10)] was used to integrate the two datasets to evaluate the conversation and variation across species for cross-species integrative analyses of human and mouse datasets. We used the function “toupper” to convert all mouse gene names into uppercase. The function “selectsGenes” (var. thresh = 0.3) was used to perform variable gene selection on human and mouse datasets separately, and then take their union. Next, we identified the cells loaded on the corresponding cell factors and quantile normalized their factor loading across datasets. The cell dimensionality reduction was performed using the function “runUMAP.” The function “plotGeneLoadings” was used to visualize the most highly loading genes (both shared and dataset specific) for each factor.

**References**

1. Butler A, Hoffman P, Smibert P, Papalexi E, Satija R (2018) Integrating single-cell transcriptomic data across different conditions, technologies, and species. Nature Biotechnology 36:411-420 doi:10.1038/nbt.4096

2. Chen C, Chen H, Zhang Y, Thomas HR, Frank MH, He Y, Xia R (2020) TBtools: An Integrative Toolkit Developed for Interactive Analyses of Big Biological Data. Mol Plant 13:1194-1202 doi:10.1016/j.molp.2020.06.009

3. Farbehi N, Patrick R, Dorison A, Xaymardan M, Janbandhu V, Wystub-Lis K, Ho JW, Nordon RE, Harvey RP (2019) Single-cell expression profiling reveals dynamic flux of cardiac stromal, vascular and immune cells in health and injury. Elife 8 doi:10.7554/eLife.43882

4. Forte E, Skelly DA, Chen M, Daigle S, Morelli KA, Hon O, Philip VM, Costa MW, Rosenthal NA, Furtado MB (2020) Dynamic Interstitial Cell Response during Myocardial Infarction Predicts Resilience to Rupture in Genetically Diverse Mice. Cell Rep 30:3149-3163 e3146 doi:10.1016/j.celrep.2020.02.008

5. Jin S, Guerrero-Juarez CF, Zhang L, Chang I, Ramos R, Kuan CH, Myung P, Plikus MV, Nie Q (2021) Inference and analysis of cell-cell communication using CellChat. Nat Commun 12:1088 doi:10.1038/s41467-021-21246-9

6. Qiu X, Hill A, Packer J, Lin D, Ma Y-A, Trapnell C (2017) Single-cell mRNA quantification and differential analysis with Census. Nature Methods 14:309-315 doi:10.1038/nmeth.4150

7. Qiu X, Mao Q, Tang Y, Wang L, Chawla R, Pliner HA, Trapnell C (2017) Reversed graph embedding resolves complex single-cell trajectories. Nature Methods 14:979-982 doi:10.1038/nmeth.4402

8. Stuart T, Butler A, Hoffman P, Hafemeister C, Papalexi E, Mauck WM, III, Hao Y, Stoeckius M, Smibert P, Satija R (2019) Comprehensive Integration of Single-Cell Data. Cell 177:1888-1902.e1821 doi:10.1016/j.cell.2019.05.031

9. Trapnell C, Cacchiarelli D, Grimsby J, Pokharel P, Li S, Morse M, Lennon NJ, Livak KJ, Mikkelsen TS, Rinn JL (2014) The dynamics and regulators of cell fate decisions are revealed by pseudotemporal ordering of single cells. Nat Biotechnol 32:381-386 doi:10.1038/nbt.2859

10. Welch JD, Kozareva V, Ferreira A, Vanderburg C, Martin C, Macosko EZ (2019) Single-Cell Multi-omic Integration Compares and Contrasts Features of Brain Cell Identity. Cell 177:1873-1887 e1817 doi:10.1016/j.cell.2019.05.006

11. Yokota T, McCourt J, Ma F, Ren S, Li S, Kim T-H, Kurmangaliyev YZ, Nasiri R, Ahadian S, Nguyen T, Tan XHM, Zhou Y, Wu R, Rodriguez A, Cohn W, Wang Y, Whitelegge J, Ryazantsev S, Khademhosseini A, Teitell MA, Chiou P-Y, Birk DE, Rowat AC, Crosbie RH, Pellegrini M, Seldin M, Lusis AJ, Deb A (2020) Type V Collagen in Scar Tissue Regulates the Size of Scar after Heart Injury. Cell 182:545-562.e523 doi:<https://doi.org/10.1016/j.cell.2020.06.030>

12. Yu G, Wang LG, Han Y, He QY (2012) clusterProfiler: an R package for comparing biological themes among gene clusters. OMICS 16:284-287 doi:10.1089/omi.2011.0118

13. Zhou Y, Zhou B, Pache L, Chang M, Khodabakhshi AH, Tanaseichuk O, Benner C, Chanda SK (2019) Metascape provides a biologist-oriented resource for the analysis of systems-level datasets. Nat Commun 10:1523 doi:10.1038/s41467-019-09234-6
